# Membrane-Modifying Effects of Perfluoroalkyl Substances in Model Bacterial Membranes

**DOI:** 10.1021/acsomega.5c04177

**Published:** 2025-08-26

**Authors:** Micaela Panella, Amani Rabadi, Jasmin Ceja-Vega, Jessica Said, Elizabeth Andersen, Joseph Mitchell, Jacqueline Ceja, Sunghee Lee

**Affiliations:** Department of Chemistry and Biochemistry, Iona University, 715 North Avenue, New Rochelle, New York 10801, United States

## Abstract

Understanding the interactions of per- and polyfluoroalkyl substances (PFAS) with bacterial membranes is essential for evaluating their ecological and health impacts. To mimic the diverse environments found in bacterial membranes, we constructed model membranes as bilayers, liposomes, and supported bilayers using binary lipid mixtures of 1,2-dioleoyl-*sn*-glycero-3-phosphocholine (DOPC) or 1,2-dioleoyl-*sn*-glycero-3-phosphoethanolamine (DOPE), with 1,2-dioleoyl-*sn*-glycero-3-phospho-(1′-rac-glycerol) (sodium salt) (DOPG)—all sharing the same acyl chains but differing in headgroup types and charges. Our findings demonstrate that salts of perfluorooctanoic acid (PFOA) and perfluorobutanesulfonic acid (PFBS) induce concentration- and lipid-dependent disordering effect in membranes composed of DOPC/DOPG (3:1 mol ratio) and DOPE/DOPG (3:1 mol ratio). Water permeability measurements reveal that membranes with greater hydrogen bonding capacity and curvature stress—such as those containing DOPE—exhibit more pronounced increases in permeability upon PFAS exposure, indicating heightened susceptibility to disruption by these contaminants. Differential scanning calorimetry (DSC) shows that DOPE-DOPG mixtures display a more significant decrease in phase transition temperature (*T*_m_) and enthalpy compared to DOPC-DOPG membranes. Moreover, Raman and attenuated total reflectance infrared (ATR-IR) spectroscopies reveal a greater increase in lipid acyl chain disorder in DOPE-DOPG mixtures upon PFAS exposure. Collectively, these findings indicate that PFAS salts not only increase membrane permeability but also destabilize lipid packing and phase organization, with the most pronounced disordering effects observed in membranes containing DOPE. Taken together, our results highlight the complex interplay of electrostatic, van der Waals, and hydrogen bonding interactions that govern the effects of PFAS salts on bacterial membrane properties, as revealed by their differential impacts on permeability and lipid organization.

## Graphical abstract

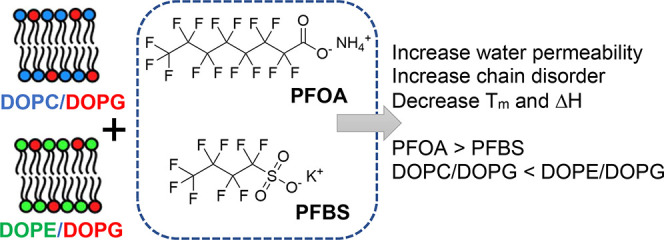

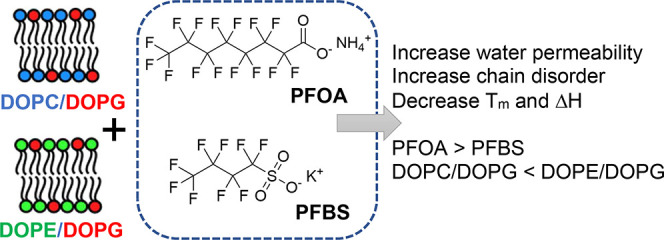

## Introduction

Per- and polyfluoroalkyl substances (PFAS) have gained significant attention due to mounting evidence of their adverse effects on human and environmental health.^1–5^ These synthetic chemicals are widely used in industrial and consumer applications, resulting in their ubiquitous distribution; and their chemical stability has led to prolonged persistence in the environment.^6–8^ For example, PFAS have been detected in groundwater and drinking water sources worldwide, and epidemiological studies have linked exposure to a range of health risks, including immunotoxicity and metabolic disruption.^1,3,5,9^

While PFAS are recognized as major environmental contaminants with significant potential to bioaccumulate in food chains and ecosystems, potentially posing threats to a wide range of organisms, research has predominantly focused on their effects in eukaryotic systems.^10^ For instance, studies have documented PFAS-induced cytotoxicity, immunotoxicity, and metabolic disruption in mammalian cells and aquatic organisms.^3,5,10–12^ In contrast, comparatively fewer studies have investigated PFAS impacts on prokaryotic organisms such as bacteria, highlighting a critical gap in our understanding of their effects on microbial communities in diverse environments.

Given the widespread occurrence of PFAS in natural environments, it is crucial to investigate their interactions with bacterial membranes, as these interactions may significantly impact microbial communities and essential ecosystem processes.^13,14^ PFAS have been shown to alter microbial community composition and inhibit key metabolic activities, potentially impacting nutrient cycling and pollutant transformation.^15,16^ Additionally, bacteria can influence the environmental fate of PFAS through processes such as sorption, retention, and partial degradation, which may inform future bioremediation strategies.^17^ Importantly, the impact of PFAS on bacterial membranes is not limited to environmental microbiota; recent studies on the one hand highlight that PFAS exposure can disrupt the gut microbiome, with implications for host health, immune function, and disease susceptibility;^18,19^ and on the other hand, an even more recent study demonstrates that human gut bacteria can bioaccumulate PFAS and accelerate their excretion.^20^ Understanding PFAS interactions with bacterial membranes at the molecular level is crucial for predicting their environmental behavior, informing risk assessment, and guiding the development of effective strategies for bioremediation and the management of PFAS contamination in water and soil.^21–24^

Research has shown that PFAS can readily penetrate zwitterionic phospholipid bilayer membranes, disrupt lipid–lipid interactions, and induce structural disorder within the bilayer.^25^ Building on this growing body of evidence regarding PFAS effects on zwitterionic phospholipid bilayers, recent studies have expanded to explore how these compounds interact with the more complex architecture of bacterial membranes.^26–28^ Although not as extensively studied as mammalian system, the effects of PFAS on bacterial membranes have garnered increasing attention.^23^ For example, studies have shown that PFAS significantly affect bacterial membrane properties, altering their integrity and functionality. These interactions can increase membrane permeability in various bacterial species, potentially leading to enhanced PFAS uptake and cellular stress responses.^26,27,29^ Moreover, the tendency of PFAS to accumulate in bacterial cells increases with the number of fluorinated carbon atoms in their structure. This accumulation is more pronounced for perfluorinated sulfonates compared to perfluorinated carboxylates.^26^

The effects of PFAS on bacteria extend beyond membrane integrity, influencing metabolic pathways and survival strategies. Exposure of *Escherichia coli* to PFAS has been linked to destabilizing its cellular membranes, inducing oxidative stress, and damaging DNA, leading to bacterial dysfunction or death.^30^ It has been reported that bacterial species exhibit varied responses and sensitivities when exposed to different PFAS, with each bacterial strain demonstrating unique interactions and metabolic adaptations to specific PFAS molecular structures.^26,31^ While some species show resilience to PFAS exposure, suggesting potential adaptation or bioaccumulation, others are more sensitive, altering community dynamics in affected ecosystems.^8,17^

To investigate the molecular interactions between PFAS and bacterial membranes, we constructed model membranes—including bilayers, liposomes, and supported bilayers—using binary lipid mixtures of 1,2-dioleoyl-*sn*-glycero-3-phosphocholine (DOPC) or 1,2-dioleoyl-*sn*-glycero-3-phosphoethanolamine (DOPE), with 1,2-dioleoyl-*sn*-glycero-3-phospho-(1′-rac-glycerol) (sodium salt) (DOPG). All three lipids share identical acyl chains but differ in headgroup types and charges, allowing us to assess the impact of lipid composition on membrane properties.

We employed DOPE/DOPG and DOPC/DOPG mixtures at a 3:1 molar ratio to systematically probe PFAS-membrane interactions. The DOPE/DOPG mixture closely mimics the lipid profile of Gram-negative bacterial membranes such as *E. coli*, where PE is the dominant phospholipid (∼70–80%) and PG provides essential negative charge (∼20–25%).^32^ Although DOPE is a nonbilayer-forming lipid, its combination with DOPG yields a stable lamellar phase under ambient conditions.^33^ The 3:1 molar ratio was chosen to maintain sufficient negative charge from DOPG while ensuring bilayer stability and reproducibility, closely matching the zwitterionic-to-anionic lipid balance found in the *E. coli* plasma (inner) membrane. Notably, our model does not incorporate lipopolysaccharides (LPS) and is specifically designed to mimic the inner (plasma) membrane, not the outer membrane of Gram-negative bacteria.^34^ The DOPC/DOPG mixture serves as a stable, zwitterionic reference, enabling direct comparison of how headgroup chemistry and membrane curvature affect PFAS interactions.

Although POPE/POPG mixtures are widely used to mimic native bacterial membranes,^32^ we selected DO-tailed lipids (DOPE, DOPG) to ensure optimal membrane fluidity and experimental reproducibility. This choice is particularly advantageous for differential scanning calorimetry (DSC), as PO-tailed lipids (e.g., POPC, POPG) can interfere with water freezing to complicate phase transition analysis, whereas DO-based lipids yield well-resolved and reproducible thermograms under our experimental conditions. Furthermore, DO-lipids remain fluid at room temperature, which is essential for our water permeability assays. Thus, our model system balances biological relevance with experimental reliability and provides a reliable platform for studying PFAS-membrane interactions.

The two perfluorinated compounds in our study—perfluorooctanoic acid salt (PFOA) and perfluorobutanesulfonic acid salt (PFBS)—differ primarily in their molecular structure: PFOA features a longer eight-carbon perfluorinated chain terminated with a carboxyl group, while PFBS has a shorter four-carbon chain with a sulfonate group at its terminus (Figure 1). These structural variations enable us to examine how PFAS chain length and terminal functional groups modulate interactions with membranes of diverse lipid compositions, exploring the poorly understood impact of individual lipid components.

**Figure 1:**
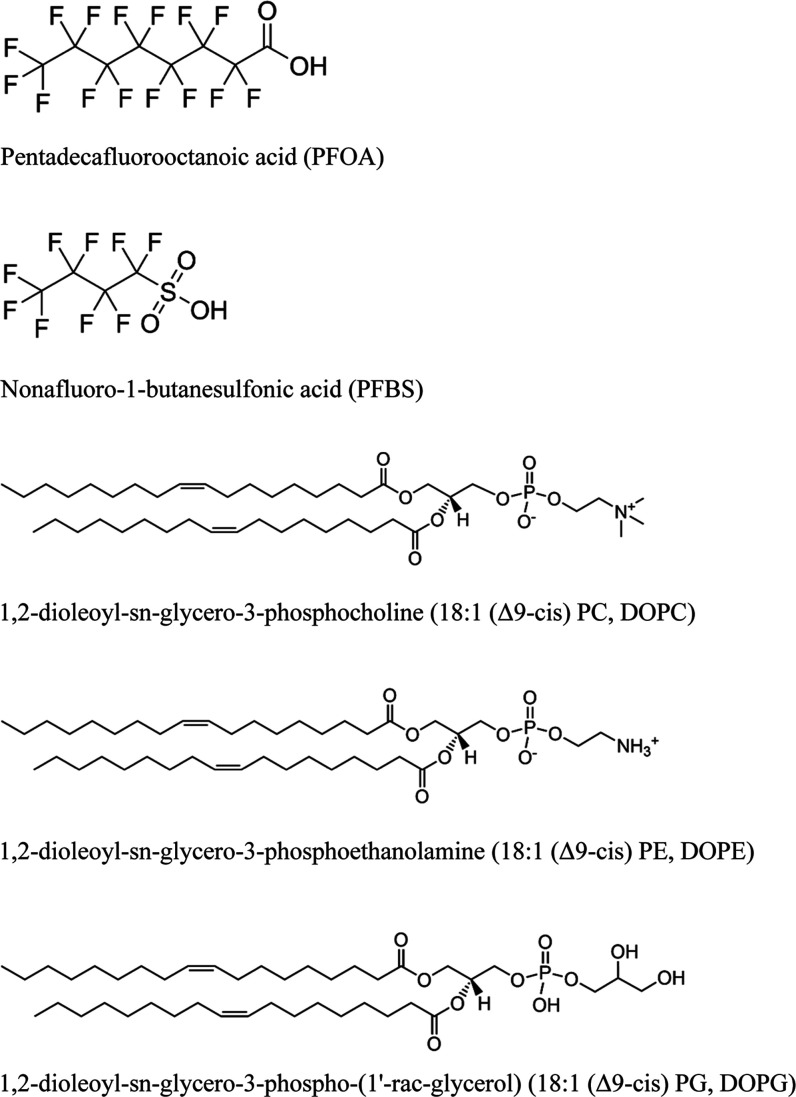
Molecular structures of PFOA, PFBS, DOPC, DOPE, and DOPG used in this study.

Maintaining membrane integrity is crucial for cellular physiology and homeostasis, as water transport across biological membranes underpins essential biological functions.^35^ Disruptions in membrane organization can compromise barrier function and alter permeability, with significant implications for cellular function.^35^ To assess how PFAS compounds such as PFOA and PFBS affect membrane integrity, we focus on their potential influence on water transport across model bacterial membranes.

We have previously developed a novel approach using droplet interface bilayers (DIBs) to investigate the impact of membrane composition on water permeability in model systems.^36,37^ DIBs are formed when oil-surrounded, lipid-monolayer-covered aqueous microdroplets are brought into contact, creating a bilayer at their interface.^38^ This self-assembled structure at the interdroplet contact zone closely resembles the double-leaflet lipid bilayer of cellular membranes and provides a versatile platform for biomimetic modeling and nanoscale chemical exploration.^37,39,40^ Our DIB-based water permeability assessments have demonstrated that water transport through a single unsupported bilayer is highly responsive to the physical state of model membranes,^37^ enabling the detection of subtle alterations in membrane composition and structure,^41,42^ as well as the effects of bioactive molecules.^43–45^

To gain further insight into the interplay between bacterial model membranes and PFAS, we employed additional techniques including differential scanning calorimetry (DSC) and ATR-IR spectroscopy of liposome suspensions, and confocal Raman microspectroscopy of supported lipid bilayers. Together, these methods provide a comprehensive evaluation of how PFAS exposure alters membrane physical properties and organization.

## Materials and Methods

### Sample Preparations

The lipids used in this study were sourced from Avanti Polar Lipids, Inc. (Alabaster, AL), with a purity of ≥99% and employed without additional purification. Each of 1,2-dioleoyl-*sn*-glycero-3-phosphocholine (DOPC), 1,2-dioleoyl-*sn*-glycero-3-phosphoethanolamine (DOPE), and 1,2-dioleoyl-*sn*-glycero-3-phospho-(1′-rac-glycerol) (sodium salt) (DOPG) was provided as a chloroform solution. Squalene (SqE, 2,6,10,15,19,23-hexamethyl-2,6,10,14,18,22-tetracosahexaene) was chosen for its ability to form essentially solvent-free droplet interface bilayers (DIBs).^46^ PFOA as its ammonium salt and PFBS as its potassium salt were obtained from Sigma-Aldrich with the highest available purity. All lipids were stored at −20 °C, whereas SqE was stored at 2–8 °C. Samples were prepared fresh immediately prior to use. For water permeability experiments, oil solutions containing binary mixtures of DOPE, DOPC, and DOPG were prepared. To do this, the chloroform solution of lipid mixture was evaporated under inert gas to create a dried lipid film, which was then subjected to overnight vacuum drying. Following this step, SqE was added to the dried film to achieve a final lipid concentration of 5 mg/mL. When used in water permeability experiments, PFOA or PFBS were added to the aqueous phase of the droplets. For differential scanning calorimetry (DSC) and ATR-IR spectroscopy, dried lipid films were rehydrated with aqueous PFOA or PFBS solutions to a total lipid concentration of ∼16 mg/mL. To form multilamellar vesicles (MLVs), the mixture was vortexed for 5 min, followed by 30 min of bath sonication. For Raman microspectroscopy, these MLVs were subjected to seven additional freeze–thaw cycles using liquid nitrogen. All aqueous solutions were prepared with deionized water (18.2 MΩ·cm) purified by a Millipore Direct Q-3 system. The osmolality of the solutions was measured using a VAPRO model 5600 vapor pressure osmometer immediately after preparation and before use.

### Differential Scanning Calorimetry

Thermal phase transition studies were performed using a TA Q2000 Differential Scanning Calorimeter (DSC). The phase transition characteristics of the samples were analyzed with TA Universal Analysis software. The main phase transition temperature (*T*_m_) was identified as the temperature at the peak of the endothermic transition, while the phase transition enthalpy (Δ*H*) was calculated by integrating the area under the heat capacity curve. Approximately 15 μL of multilamellar vesicles (MLVs), prepared as described in the sample preparation section, were sealed in DSC pans and subjected to heating and cooling cycles at a rate of 5 °C/min between −38 °C and −2 °C under a high-purity nitrogen atmosphere flowing at 50 mL/min. To ensure reproducibility and assess potential hysteresis effects, each sample underwent three consecutive cycles, yielding consistent results across all cycles. The reported values represent the average of measurements from at least three independently prepared samples, expressed as mean ± standard deviation. Additionally, baseline subtraction and curve-fitting simulations were conducted using OriginPro 10.1 software to deconvolute the data into multiple components, each corresponding to distinct *T*_m_ regions.

### Attenuated Total Reflectance-Fourier Transform Infrared Spectroscopy (ATR-FTIR)

ATR-FTIR spectral analysis was performed using a Thermo Scientific Nicolet iS20 spectrometer equipped with a deuterated triglycine sulfate (DTGS) detector. The measurements utilized a GladiATR single-reflection ATR accessory featuring a diamond crystal and a temperature-controlled plate (Pike Technologies, USA). For each measurement, approximately 40 μL of multilamellar vesicles (MLVs), prepared as described in the experimental section, were applied to the diamond crystal surface. An ATR liquid retainer and volatiles cover accessory (Pike Technologies, USA) were used to contain the sample. Spectra were collected over the 400–4000 cm^–1^ range at 25 °C, with 200 scans accumulated at a spectral resolution of 4 cm^–1^. Background spectra of deionized water were recorded prior to each new sample and subtracted from the sample spectra. Following each measurement, the diamond crystal, ATR liquid retainer, and volatiles cover were thoroughly cleaned with isopropanol and allowed to dry before the next sample. To ensure reproducibility, three independent sets of samples were prepared, with each sample scanned twice for consistency. The ATR-FTIR data represent the average of all samples and are expressed as mean ± standard deviation. Data processing was conducted using OMNIC 9 software (Thermo).

### Confocal Raman Microspectroscopy

Raman spectroscopic experiments were performed using an inverted confocal Raman microscope system (XploRA INV, Horiba) which consists of a Raman spectrometer directly coupled to an inverted microscope (Nikon Eclipse Ti–U). The Raman setup includes an internal laser kit operating at 532 nm (air-cooled solid-state laser) and a thermoelectrically cooled CCD detector. A 10× microscope objective was used for focusing a 532 nm wavelength laser beam, and for collecting Raman scattered light, subsequently dispersed with a grating of 1800 lines per millimeter. The glass coverslips (#1.5) used as substrates for deposition of lipid bilayer films were rinsed with ethanol and blown dry with N_2_ prior to use. A sample (10 to 20 μL) of lipid mixture suspension, immediately after generation by a freeze–thaw process (as described in sample preparation section), was spread on the surface of the cleaned coverslip and the aqueous solvent was allowed to evaporate in a closed homemade chamber on top of a heating plate at ∼30 °C, to form a solid supported lipid bilayer on a hydrophilic surface. All Raman spectra described herein are obtained at ambient room temperature, ca. 25 °C. Three independent samples were prepared, and each was scanned across multiple regions. The reported values represent the average of these measurements.

### Water Permeability Measurement Using Droplet Interface Bilayer (DIB)

Water permeability measurements were conducted using model membranes formed by the droplet interface bilayer (DIB) method. The general experimental setup and procedure have been described in detail elsewhere.^41^ The setup consists of an inverted microscope (Nikon Eclipse Ti–S with halogen lamp) combined with two hydraulic micropipet manipulators (Narishige), all supported on a vibration-isolated workstation (Newport). A camera (Andor Zyla sCMOS) is directly attached to the microscope for real-time recording of the generated microdroplets and their size changes. Glass micropipets with tapered ends, controlled by the manipulators, were used to dispense aqueous microdroplets into oil. Two osmotically unbalanced aqueous droplets, each approximately 100 μm in diameter, were dispensed into a squalene solvent containing lipids. One droplet contained pure water, while the other contained 0.1 M NaCl with a given concentration of PFOA or PFBS. All water permeability experiments were carried out at 30 °C using a custom-built temperature-controlled microchamber, which was thermostated via an external circulating water bath. Water permeability data represent an average of individual permeability runs (*n* ≥ 50), and standard deviation as error bars (mean ± standard deviation). The recorded videos and images were postanalyzed to measure the dimension of droplets and bilayer contact area, using custom built image analysis software. The detailed methodology for determining water permeability coefficient is provided in the Supporting Information.

## Results and Discussion

### Effect of PFOA and PFBS on Barrier Properties of Bacterial Model Membranes

Figure 2A shows a general schematic of a DIB formed by binary mixtures of DOPC or DOPE, with DOPG, in SQE. When there is an osmotic pressure difference between two adjacent aqueous microdroplets in a DIB, water moves across the DIB, resulting in a measurable change in the diameter of the droplets, as illustrated schematically in Figure 2A. Figure 2B,C present the relative osmotic water permeability coefficients ( ${P_{f}/P}_{f}^{o}$ where *P*_f_^o^ represents the osmotic water permeability in the absence of PFAS) of lipid mixtures at 30 °C, illustrating the impact of molecules PFOA and PFBS on water permeability across two mixed lipid membranes, DOPC/DOPG (3:1 mol ratio) and DOPE/DOPG (3:1 mol ratio). The corresponding numerical values for permeability coefficients are provided in Table S1 (Figure S1 in Supporting Information). Statistical comparisons between each concentration and the control were performed using *t* tests. A *p*-value less than 0.05 was considered statistically significant, and significance is indicated in the Figure 2 legend. Additional statistical comparisons between successive concentration values are indicated in the Supporting Information (Table S1).

**Figure 2:**
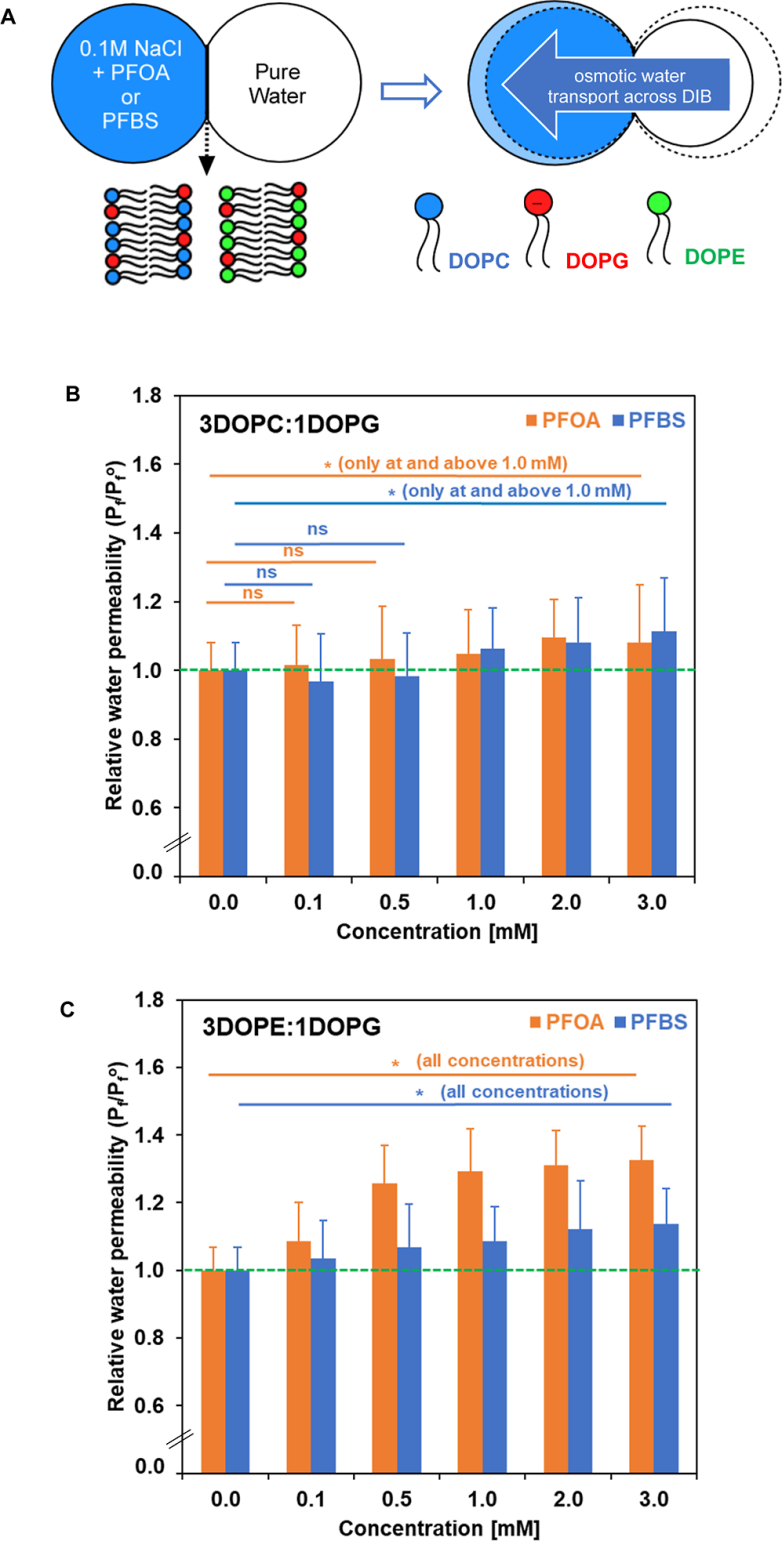
(A) General schematic of osmotic water permeability measurement using DIB formed by binary lipid mixtures of DOPE, DOPG, and DOPC; and relative osmotic water permeability coefficients (*P*_f_/*P*_f_^o^) of lipid bilayer formed from lipid mixtures of (B) DOPC/DOPG (3:1) and (C) DOPE/DOPG (3:1) at 30 °C with varying concentrations of PFOA (orange column) or PFBS (blue column). The dotted green horizontal line shows a control value. All values are normalized to their respective control means. Error bars for the control represent the relative standard deviation of the raw control measurements prior to normalization. Statistical analyses were performed using *t* tests. Significance versus control are indicated as “*” for a *p*-value less than 0.05, or ns for *p*-value greater than 0.05.

The results from Figure 2B and Table S1 reveal that for membrane DOPC/DOPG (3:1), water permeability at lower concentrations (0.1–0.5 mM) showed generally nonsignificant effects, with the statistically significant increases relative to control appearing at and above 1.0 mM concentrations of PFOA and PFBS. At a 3 mM PFAS concentration, membrane with PFOA and PFBS shows 67 ± 9 μm/s and 69 ± 8 μm/s, respectively, compared to the control 62 ± 5 μm/s for this lipid mixture. In contrast, membrane DOPE/DOPG (3:1) displays more substantial changes in water permeability with increasing concentrations of PFOA and PFBS, as seen in Figure 2C. Exposure of model membranes of DOPE/DOPG (3:1) to increasing concentrations of PFAS resulted in concentration-dependent increase in water permeability, with statistically significant differences observed at all concentrations compared to control (*p* < 0.05). The effect is notably more prominent for PFOA. At 3 mM PFAS concentration, the DOPE/DOPG membrane with PFOA reaches 77 ± 2 μm/s, and the membrane with PFBS increases to lesser extent to 66 ± 4 μm/s, in comparison to its control value of 58 ± 4 μm/s. For both model membranes, increase in water permeability plateaued at higher concentrations, indicating saturating effects. Note that the differing values for the respective controls (Table S1 in the Supporting Information) are a function their lipid mixtures. The incorporation of negatively charged DOPG into DOPC is reported to result in a decrease in area per molecule and an increase in bilayer thickness;^47^ such a compacted bilayer structure is reflected in a reduced water permeability of 62 ± 5 μm/s for membranes of DOPC/DOPG (3:1) compared to a pure DOPC bilayer (70–75 μm/s at 30 °C).^43,48^ In membrane mixtures of DOPE and DOPG (3:1 mol ratio), the water permeability value is shown to be 58 ± 4 μm/s (Table S1). DOPE has a smaller headgroup than DOPC,^49^ leading to generally tighter packing. Consequently, the water permeability coefficient for DOPE/DOPG is expected to be lower than those for DOPC/DOPG, as shown in our study.

Overall, the changes in water permeability differ significantly based on the specific lipid mixture composition, highlighting the complex interactions between molecules PFOA and PFBS with different membrane structures. Water permeability across lipid bilayers is governed by various structural and physical properties of both individual lipids and the bilayers they compose. The thickness of the bilayer, the molecular area each lipid occupies, and the overall fluidity of the membrane are key factors influencing water transport.^50–52^ Membrane fluidity, which is closely related to the packing density of lipids, plays a crucial role in determining water permeability.^53^ It is generally expected that tighter lipid packing within the bilayer region leads to reduced water permeability, while looser packing may facilitate easier water passage through the membrane. Our experimental results, which demonstrate an overall increase in water permeability upon exposure to PFAS molecules, suggest that the presence of these molecules influences the structural integrity of the membrane environment. This perturbation likely compromises the intermolecular interactions between lipid headgroups and bilayer packing. Consequently, these changes may lead to enhanced membrane fluidity, which could explain the observed increase in water permeability across the lipid bilayer.

The more substantial increase in transbilayer water permeability observed in lipid mixtures containing DOPE compared to DOPC (having the same incorporation of DOPG) suggests a higher degree of membrane perturbation caused by these molecules, particularly molecule PFOA. Molecular dynamics (MD) simulations have demonstrated that hydrogen bonding interactions between PFAS molecules and lipid components serve as one of the key pathways facilitating PFAS penetration into lipid membranes.^54^ The differential interactions between lipid mixtures containing DOPE and DOPC can be attributed to their hydrogen bonding capabilities with PFAS. DOPE exhibits stronger hydrogen bonding due to the presence of its amine (NH_3_^+^) group, which acts as a hydrogen donor. In contrast, DOPC, with its headgroup dominated by a choline moiety containing three methyl groups attached to the nitrogen atom, is less capable of forming hydrogen bonds compared to DOPE. Consequently, DOPE-containing membranes are more accessible for binding with PFAS, and the enhanced perturbation likely results in a more significant impact on membrane fluidity, as evidenced by the greater changes in water permeability. In addition, it is important to note that PC lipids have near-zero intrinsic curvature, while PE lipids have negative intrinsic curvature.^55^ As a result of this difference, DOPE tends to induce negative curvature in membranes due to its conical shape, which can create areas of increased stress and reactivity. This curvature stress can make the membrane more susceptible to interactions with PFAS, compared to DOPC which forms a bilayer with less curvature owing to its cylindrical molecular structure.

The prominent effect on water permeability for PFOA with DOPE/DOPG (3:1) membranes, relative to PFBS (as noted above), is likely a function of the relative hydrophobicities of the respective PFAS. The greater membrane/water partition coefficients^56^ observed for PFOA (log *K*_mem/w_ = 3.52) relative to PFBS (log *K*_mem/w_ = 2.86) underscore the importance of hydrophobic interactions in determining how long-chain and short-chain PFAS molecules interact with membranes and influencing the barrier properties of membranes.

Our observation of PFAS-induced increasing water permeability is qualitatively consistent with previous studies. For example, PFAS exposure has been shown to enhance the microbial quorum sensing response in the model bacterium *Aliivibrio fischeri*, which was attributed to increased membrane permeability resulting from PFAS interaction.^27^ The monolayer studies of DMPC, DMPE, and DMPG in the presence of various PFAS molecules also show an overall greater susceptibility for DMPE on its surface pressure-mean molecular area isotherms.^57^ The interaction mechanisms of PFOA or PFBS with membranes containing different lipid components are complex and can involve multiple forces, including electrostatic interactions, van der Waals forces, and hydrogen bonding. These interactions collectively influence the packing characteristics of mixed lipid membranes, which can be observed in the changes in water permeability values, as demonstrated here. Moreover, the differing impacts on water permeability observed with PFOA and PFBS underscore how sensitive these molecules are to variations in their molecular structures and the lipid compositions.

### Phase Transition Behavior of Bacterial Model Membranes upon Exposure to PFOA and PFBS

The endothermic differential scanning calorimetry (DSC) thermograms and their associated thermodynamic data for multilamellar vesicles (MLVs) composed of lipid mixtures, are displayed in Figures 3 and 4. An expanded region is also shown in each figure, highlighting the thermogram at high concentrations of PFAS. The corresponding thermodynamic data are listed in Table S2. These results illustrate the effects of varying concentrations of PFOA and PFBS on the thermal behavior of the lipid systems. The DSC thermogram of pure DOPC/DOPG at 3:1 molar ratio MLVs (Figure 3 and Table S2A) reveals a well-defined endothermic transition at approximately ∼*T*_m_ = −17 °C, with an associated enthalpy of about 8.5 kcal/mol. Binary mixtures of PG and PC with identical acyl chains exhibit a narrow melting range, suggesting complete miscibility and similar transition temperatures and enthalpies between the two components^58^ (the transition temperatures (*T*_m_) and transition enthalpies (Δ*H*) of DOPC are −18.3 ± 3.6 °C and 9.0 ± 2.8 kcal/mol).^59^ This transition represents the phase change from the lamellar gel phase *L*_β_ to the lamellar liquid-crystalline state *L*_α_. The relatively low *T*_m_ of the mixture of DOPC/DOPG indicates that its membrane is in a disordered, fluidic state at ambient temperatures, a characteristic typically associated with having two monounsaturated acyl chains. Figure 3 and Table S2A reveal that, while PFOA and PFBS have minimal impact on the transition temperature (*T*_m_) of lipid mixture DOPC/DOPG, they induce a concentration-dependent reduction in transition enthalpies, accompanied by substantial peak broadening. When PFOA is incorporated into the bilayer at a 4:1 mol ratio of lipid mixtures to PFOA (the highest concentration we tested), the *T*_m_ decreases by approximately 0.7 °C compared to the control, accompanied by a reduction in enthalpy (Δ*H*) from 8.35 (control) to 2.86 kcal/mol. The proportion of PFAS in multilamellar vesicles is assumed from the molar ratios of total lipid to PFAS (lipid/PFAS) used to formulate the liposome suspension. The presence of PFBS at the same ratio results in a slightly smaller decrease in *T*_m_ of approximately 0.3 °C, with a change in Δ*H* from 8.66 (control) to 4.52 kcal/mol. At this concentration, the full width at half-maximum (fwhm) values for the peaks are 4.17 °C for PFOA and 3.46 °C for PFBS, compared to 0.46 °C-0.52 °C for the control.

**Figure 3:**
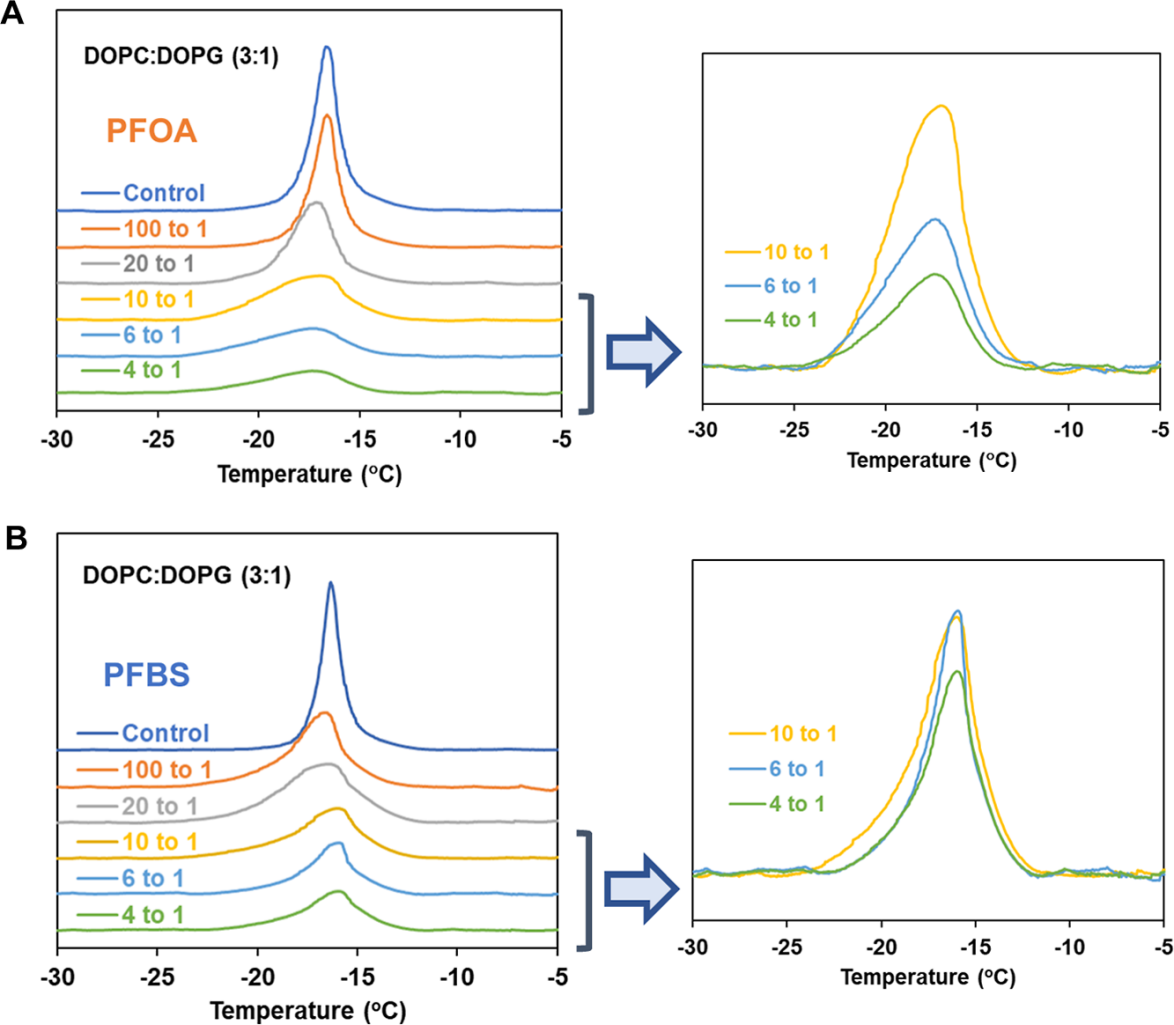
Endothermic calorimetric thermograms of DOPC/DOPG (3:1) MLVs containing (A) PFOA and (B) PFBS with varying concentrations.

**Figure 4:**
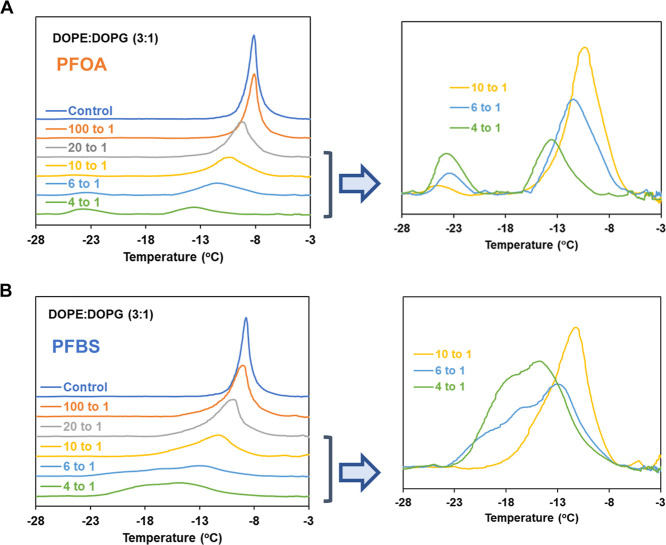
Endothermic calorimetric thermograms of DOPE/DOPG (3:1) MLVs containing (A) PFOA and (B) PFBS with varying concentrations.

Model membranes consisting of the lipid mixture DOPE/DOPG (3:1 molar ratio) form stable bilayers at ambient temperature and produce well-defined endothermic transition temperature approximately at −8 to −9 °C with transition enthalpies of ∼7–8 kcal/mol (Figure 4 and Table S2B), again representing the phase changes from the lamellar gel phase *L*_β_ to the lamellar liquid-crystalline state *L*_α_. The reported thermodynamic parameters (the lamellar gel phase *L*_β_ to the lamellar liquid-crystalline state *L*_α_) for pure DOPE is −7.34 ± 3.78 °C.^60^ Both PFOA and PFBS exhibit a significant concentration-dependent effect on the lipid mixture DOPE/DOPG, manifesting as a reduction in both transition temperature (*T*_m_) and transition enthalpies (Δ*H*). These changes are accompanied by substantial peak broadening. At higher concentrations, the peak separates into two distinct components with different peak temperatures, as illustrated in Figure 4 and Table S2B. To analyze this complex behavior, curve-fitting simulations were employed to deconvolute the data into two components, corresponding to lower and higher *T*_m_ regions. These regions likely represent differential incorporation of PFAS into the lipid mixtures (Figure S2 in the Supporting Information). Note that for thermograms exhibiting multiple peaks, the total area across all peaks was used to calculate the enthalpy data.

For relative comparison, Figure 5A,B shows the changes in main phase transition temperature (*T*_m_ – *T*_m_°), where *T*_m_ and *T*_m_° are the main phase transition temperatures in the presence and absence of given concentrations of PFAS, respectively, plotted as a function of mole fraction of PFAS for DOPC/DOPG (3:1) (Figure 5A) and DOPE/DOPG (3:1) (Figure 5B). Analogously, Figure 5C,D plots the changes of the enthalpy of transition (shown as the ratio Δ*H*/Δ*H*°) as a function of the mole fraction of PFAS for DOPC/DOPG (3:1) (Figure 5C) and DOPE/DOPG (3:1) (Figure 5D), where Δ*H* and Δ*H*° are the transition enthalpies in the presence and absence of PFAS, respectively. Figure 5A–D reveals that while PFOA and PFBS induce negligible changes in transition temperature shifts (*T*_m_ – *T*_m_°) for DOPC/DOPG (3:1) mixtures, they produce more dramatic effects in DOPE/DOPG (3:1) mixtures, causing a concentration-dependent decrease in transition temperature. However, the impacts of PFOA and PFBS on changes in transition enthalpies and peak broadening are generally comparable within the bounds of standard deviation for both lipid systems (Figure 5C), indicating the disruption of the acyl chain packing for both lipid model systems. For DOPE/DOPG membranes, the changes of the enthalpy of transition exhibit a more pronounced reduction when exposed to PFOA compared to PFBS (Figure 5D). Our DSC data corroborates the water permeability findings, demonstrating that DOPE-containing membranes, despite having the same negative charge as DOPC-containing membranes, exhibit greater susceptibility to PFAS exposure, presumably due to the greater hydrogen bonding capability of PE headgroup and curvature stress and reactivity, as discussed in the previous section.

**Figure 5:**
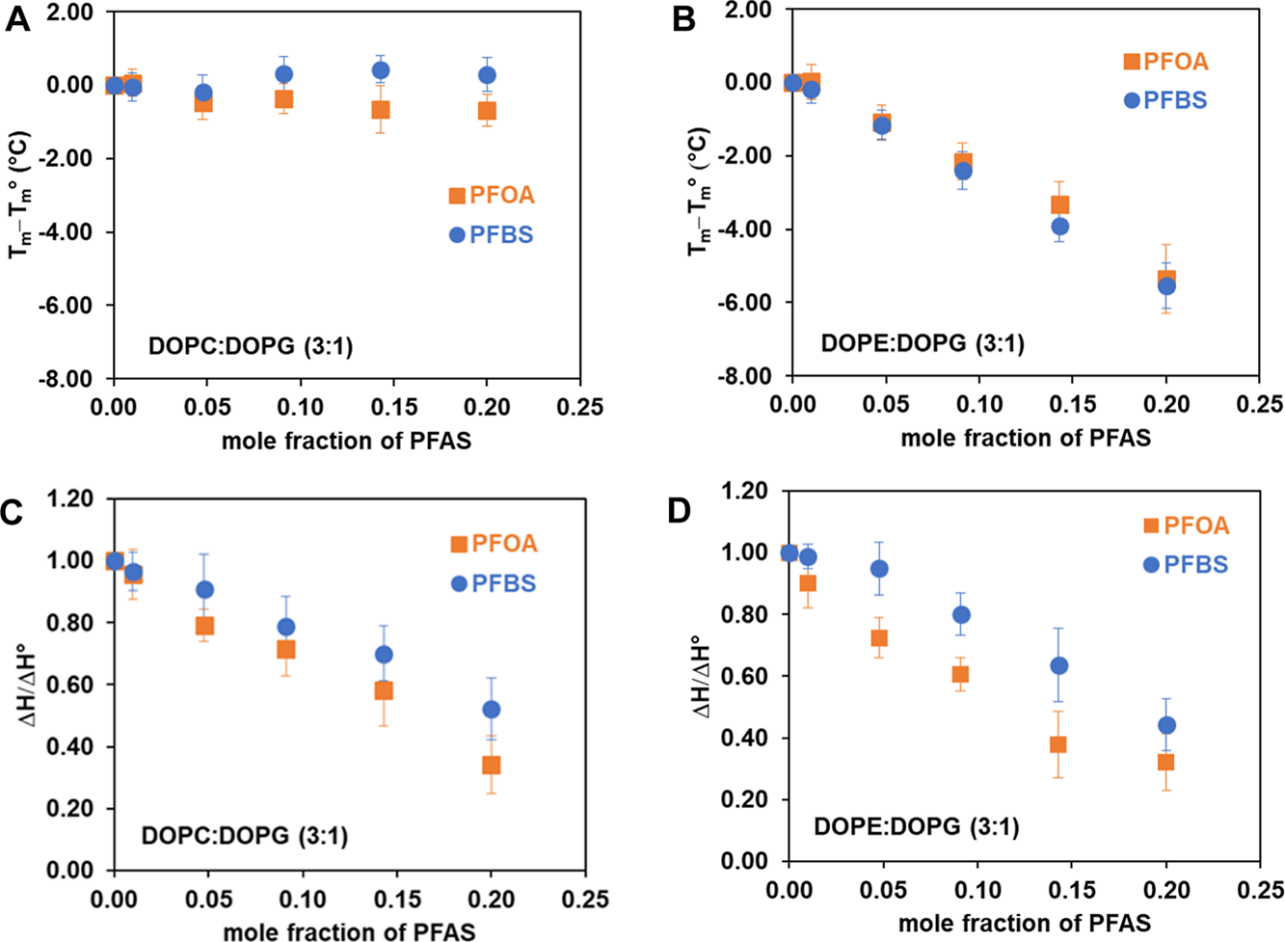
Comparison of the effect of PFOA and PFBS on the relative main transition temperature for (A) DOPC/DOPG (3:1) and (B) DOPE/DOPG (3:1), and the relative enthalpy for (C) DOPC/DOPG (3:1) and (D) DOPE/DOPG (3:1) MLVs.

Our DSC results demonstrate that PFOA and PFBS interact with lipid mixtures, modifying bilayer assemblies and their thermotropic properties. These effects are modulated by both PFAS concentration and lipid composition. Our findings align qualitatively with previous DOPC/DOPG monolayer studies, which have shown that the cis double bonds in DOPC and DOPG acyl tails create fluid monolayers with lower packing densities compared to saturated lipids and that this increased fluidity provides void spaces that readily accommodate PFOA and PFOS, thereby mitigating the impact of unfavorable electrostatic repulsion.^28^ PFAS penetration into lipid assemblies disrupts the bilayer’s long-range order, leading to a decrease in transition temperature and enthalpy. These effects are likely attributed to reduced van der Waals interactions among the hydrophobic chains when PFAS molecules are present. Similar phase separations and domain formation have been reported from Brewster Angle Microscopy studies, in DOPC monolayers exposed to PFOA and PFOS.^28^

### Structural Properties of Bacterial Model Membranes upon Exposure to PFOA and PFBS

This section examines how PFOA and PFBS influence the structural characteristics of lipid mixtures, utilizing two vibrational spectroscopic methods: ATR-FTIR spectroscopy; and confocal Raman microspectroscopy.

#### ATR-FTIR Spectroscopic Studies

Figure 6 presents ATR-FTIR spectra in the CH_2_ stretching region (2800–3000 cm^–1^) for model membranes composed of DOPE/DOPG (3:1) in the presence of varying concentrations of PFOA and PFBS at 25 °C. The spectra have been vertically shifted for clarity. An expanded view of the CH_2_ antisymmetric stretch (ν_as_) region (2910–2940 cm^–1^) is also shown in Figure 6 to highlight peak shifts. There was no observable change in the symmetric stretching (ν_s_) part of the spectra. From control (zero PFAS) up to a 20:1 molar ratio (lipid mixture to PFAS), no distinguishable shifts are observed with increasing concentrations of PFOA and PFBS. At higher PFAS concentrations (at 4:1 molar ratio), small blue shifts (∼1.5 cm^–1^) in the CH_2_ antisymmetric stretching region (ν_as_) are observed in the presence of PFOA (best depicted in Figure 6A), while lesser blue shifts (∼1.0 cm^–1^) are seen with PFBS. The corresponding values are tabulated in the Supporting Information (Table S3).

**Figure 6:**
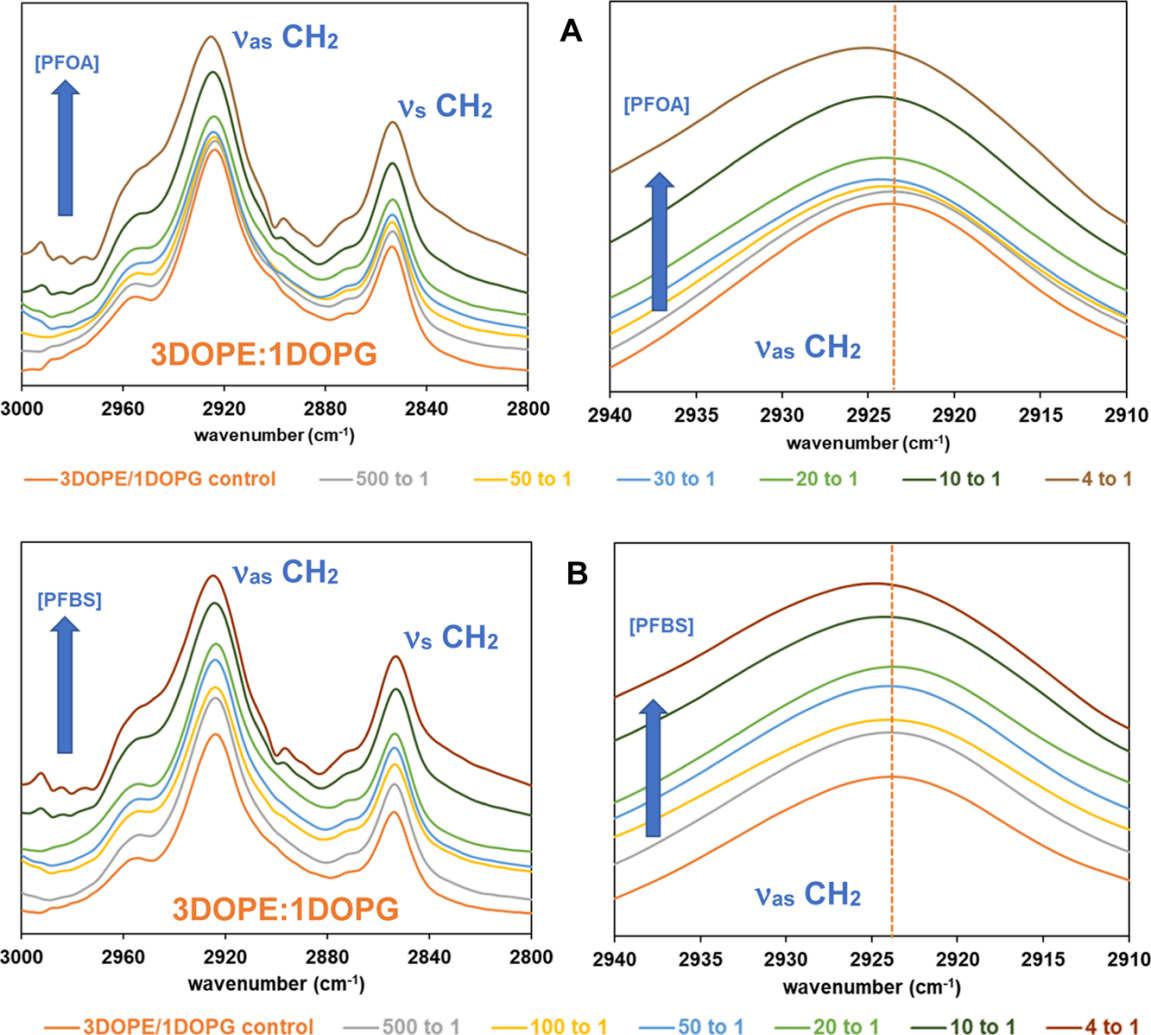
Representative ATR-IR spectra in the stretching vibration of the acyl chain CH_2_ groups of DOPE/DOPG (3:1) model membranes, with increasing concentrations (molar ratio of lipid to PFAS) of (A) PFOA and (B) PFBS at 25 °C, with the expanded regions of ν_as_ CH_2_. A vertical dotted line indicates the position of the ν_as_ CH_2_ band in the control spectrum.

The blue shift in ν_as_ CH_2_ band suggests an increase in gauche conformers and reduced acyl chain order, indicating a more disordered state of the lipid membranes upon exposure to PFOA.^61^ Our findings are qualitatively consistent with previous studies on DMPC bilayers, which reported shifts in the ν_as_ CH_2_ band toward higher frequencies (∼1–2 cm^–1^) in the presence of PFOA or PFOS.^62^ Compared to the effects seen in membranes formed from DOPE/DOPG (3:1), the observed shift in ν_as_ CH_2_ band is minimal when PFOA or PFBS are introduced to membranes formed from DOPC/DOPG (3:1) (see Figure S3 in the Supporting Information).

Phospholipid membranes also exhibit characteristic infrared-active stretching vibration regions, including the phosphate headgroup (PO_2_^–^, around 1000–1240 cm^–1^) and the interfacial carbonyl group (C=O, around 1730 cm^–1^), which can serve as potential diagnostic absorption bands for membrane structure.^63^ These regions are sensitive to hydrogen bonding and have been used for detecting structural and organizational changes in lipid bilayers upon interaction with bioactive molecules.^61,64,65^

Figure 7 displays ATR-FTIR spectra in the antisymmetric phosphate PO_2_^–^ region (1130–1290 cm^–1^) for model membranes composed of DOPE/DOPG (3:1) in the presence of varying concentrations of PFOA and PFBS at 25 °C. The bottom trace of Figure 7 also shows spectra for aqueous solutions of pure PFOA and PFBS. As seen in Figure 7, the phosphate headgroup region overlaps with C–F vibration bands (ν_as_ CF_2_ at 1243.4 cm^–1^ and ν_as_ CF_2_ + ν_as_ CF_3_ at 1210.6 cm^–1^) from PFAS,^66^ complicating the accurate analysis of these peaks, especially at the high concentrations where we detected blue shifts in ν_as_ CH_2_. Fortuitously, at very low concentrations of PFAS (up to 20:1 lipid/PFAS molar ratio), the antisymmetric PO_2_^–^ peak is discernible owing to a negligible interference from C–F bands. The antisymmetric PO_2_^–^ peak appears to undergo a red shift in the presence of PFOA (red shift of ∼5 cm^–1^ at 20:1 lipid PFOA), whereas relatively smaller changes are observed with PFBS in the same region (red shift of ∼2 cm^–1^ at 20:1 lipid PFBS). It has been well-established that changes in the wavenumber values of ν_as_ PO_2_^–^ represent the hydration profile of phospholipid headgroups.^63^ A decrease (red shift) in the wavenumber of ν_as_ PO_2_^–^ may indicate increased hydration, or a direct interaction at the phosphate headgroup (e.g., replacement of hydration water and binding to the phosphate group). Notably, a red shift in the characteristic vibrational bands of free PFOA and PFBS is observed upon their interaction with lipid membrane mixtures at 4 to 1 molar ratio of lipid to PFOA (from ν_as_ CF_2_ at 1243.4 cm^–1^ for free PFOA to 1238.1 cm^–1^ for 4 to 1 molar ratio of lipid to PFOA), further indicating the change in the molecular environment, likely due to binding of PFOA to the membranes. Additionally, the lipid C=O stretching vibration region shows a broad band around 1735 cm^–1^, with no visibly significant changes (see Figure S4 in the Supporting Information). Consistent with our observation, no changes in the C=O band have been reported when PFAS (PFOA, PFOS, PFBS) were introduced to DMPC, DMPE, DMPG, and cardiolipin monolayers, as studied by Infrared Reflection–Absorption Spectroscopy.^57^ Additionally, similar spectra for both phosphate and lipid carbonyl regions of DOPC/DOPG (3:1) membranes are provided in the Supporting Information (Figure S5). In comparison to DOPE/DOPG (3:1), DOPC/DOPG (3:1) membranes show no observable shifts in the antisymmetric PO_2_^–^ peak, even at low concentration ratios (up to 20:1). The analytical challenges at high concentrations described above, including band overlap and spectral interference, are also applicable to these membranes.

**Figure 7:**
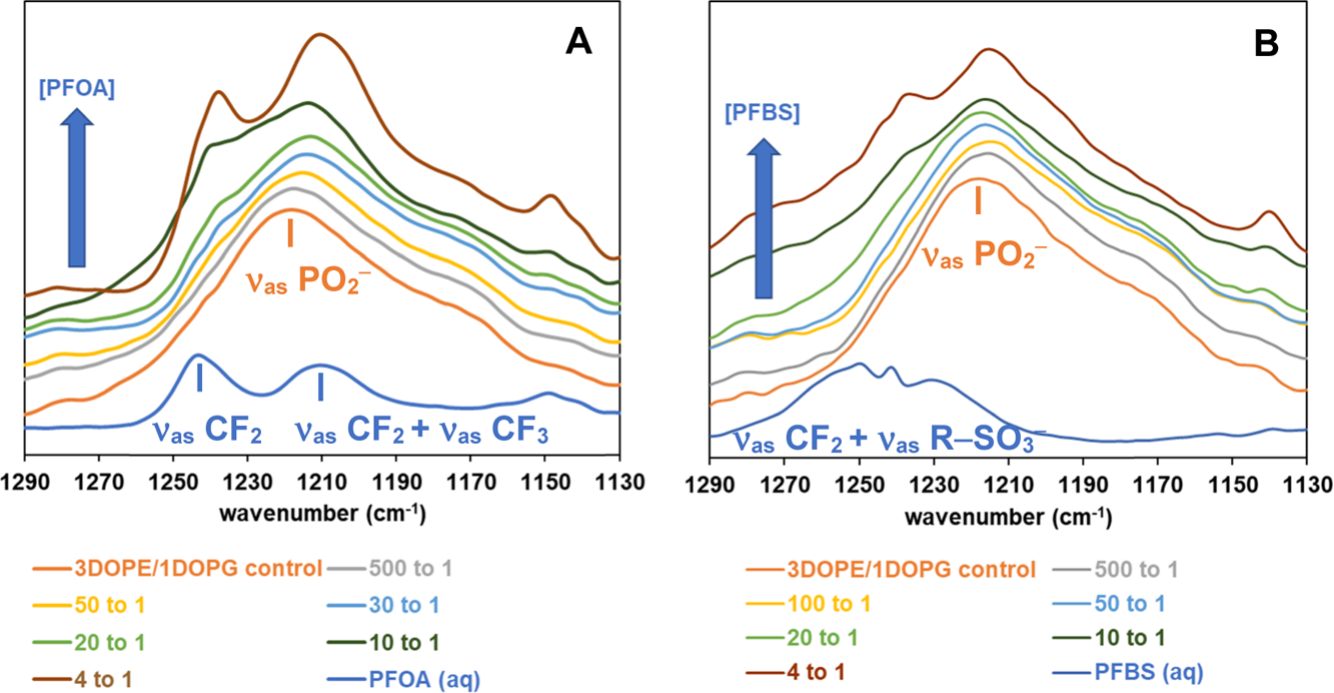
Representative ATR-IR spectra in the region of antisymmetric PO_2_^–^ stretching vibration bands of DOPE/DOPG (3:1) with varying mol ratios of (A) PFOA and (B) PFBS. The blue trace at the bottom of each panel represents the ATR-IR spectrum of PFOA or PFBS in aqueous solution.

Modifications or interactions at the phospholipid headgroup and changes in the acyl tails, can be closely coupled and significantly influence overall membrane properties.^67^ Consistent with this fundamental principle, our data and previous IR reflection–absorption spectroscopy studies indicate that PFAS—including PFBS, PFOA, and PFOS—strongly interact with phospholipid headgroups, primarily via hydrogen bonding to phosphate moieties.^57^ This interaction disrupts headgroup packing and the local electrostatic environment, weakening headgroup–headgroup associations and reducing membrane cohesion. Consequently, the acyl chains exhibit increased motional freedom and a higher proportion of gauche (kinked, disordered) conformations, manifesting as enhanced membrane fluidity.^62^ Thus, it is believed that PFAS-induced perturbations at the headgroup level indirectly promote conformational disorder in the acyl chains, resulting in a more dynamic and less rigid bilayer structure. Our findings are in agreement with IR studies showing that PFOA and PFOS increase gauche conformations in the alkyl chains of DMPC^62^ and are further supported by vibrational sum frequency generation spectroscopy, which reveals that perfluoroheptanoic acid perturbs alkyl chain ordering in DPPC monolayers on water surfaces.^68^

In summary, characteristic changes in the CH_2_ region and PO_2_^–^ vibrational regions provide evidence that both PFOA and PFBS interact with the polar headgroup region of lipids and influence the conformation of the lipid assembly in a concentration-dependent manner, with PFOA inducing more pronounced spectral shifts and conformational changes than PFBS. Notably, these effects are more substantial in DOPE/DOPG membranes compared to DOPC/DOPG membranes.

#### Raman Spectroscopic Studies

We utilized confocal Raman microspectroscopy of membrane films to investigate changes in mixed-lipid membranes upon exposure to varying concentrations of PFOA and PFBS molecules. The ambient temperature Raman spectra, spanning 600–3600 cm^–1^, are provided in the Figure 8 for DOPE/DOPG (3:1) membranes at various concentrations of PFOA. All other Raman spectra for DOPE/DOPG with PFBS, DOPC/DOPG with PFOA and PFBS as a function of concentrations are shown in the Supporting Information (Figures S6 and S7). All spectra were normalized to the intensity around 2845 cm^–1^ to facilitate comparison. Figure 8 also shows the C–H stretching region (2750–3050 cm^–1^) of the spectra, which is notable for its intense Raman scattering in phospholipid molecules and its association with hydrocarbon chain order in lipid membranes.^69,70^ Within this region, characteristic peaks for phospholipids are observed: the methylene CH symmetric stretching mode appears around 2850 cm^–1^, the antisymmetric methylene CH stretching mode near 2890 cm^–1^, and the symmetric stretching mode of the terminal methyl CH group at approximately 2930 cm^–1^. These peaks, along with their relative intensity ratios, serve as valuable markers for assessing membrane structural characteristics. Specifically, the intensity ratio between the terminal CH and symmetric CH stretching modes is sensitive to intermolecular chain coupling.^71,72^ In this study, the ratios of the peak intensities of [CH_term_ (2930)/CH_sym_ (2845)] is used as a measure of hydrocarbon chain ordering and overall packing efficiency of lipid bilayers. Figure 9 depicts relative Raman intensity ratios *I*/*I*_o_ which is affected by the presence of PFOA or PFBS, where I_o_ is the intensity ratio (2930/2845) in the absence of such molecules, and I is the intensity ratio (2930/2845) in the presence of these PFAS. The corresponding values are tabulated in the Supporting Information (Table S4). As seen in Figure 9, an increased concentration of PFAS in the mixed membranes leads to a general increase in the peak intensity ratio. The greatest change occurs with exposure of PFOA to the DOPE/DOPG membrane, while no significant differences are observed among the remaining systems.

**Figure 8:**
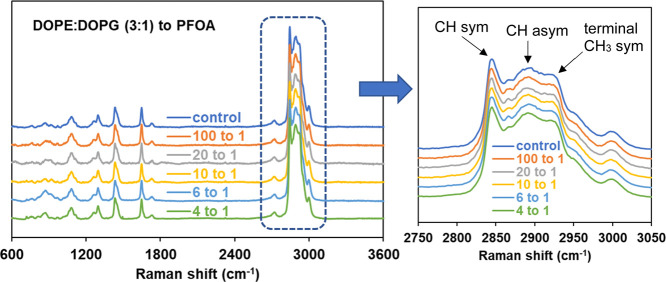
Representative Raman spectra of DOPE/DOPG (3:1) at various concentrations of PFOA at ambient temperature, and the CH stretching vibration region. Spectra are normalized to the intensity at ∼2845 cm^–1^ (the most intense peak) for comparison, and vertically shifted for clarity.

**Figure 9:**
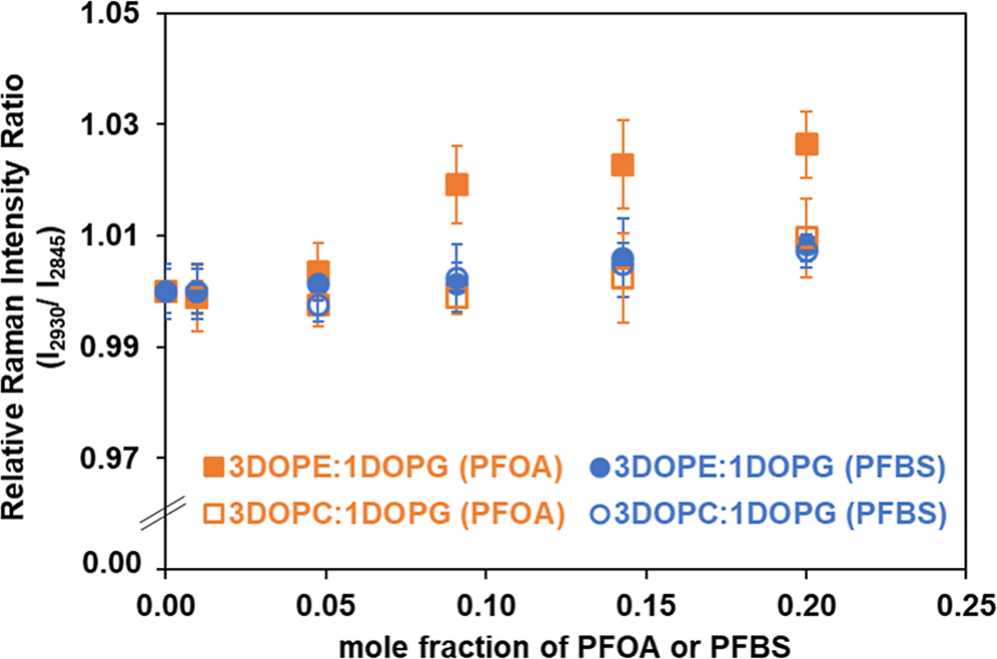
Relative Raman intensity ratios (*I*/*I*_o_) of [CH_term_ (2930)/CH_sym_ (2845)] as a function of PFOA or PFBS concentration in binary mixed membranes at ambient temperature.

In addition, the frequencies of the ∼2845 and 2890 cm^–1^ peaks represent the level of conformational order and interchain coupling in the lipid chains.^71,72^ Overall, there are small blue shifts in the CH stretching vibration frequencies of the DOPE/DOPG mixed membranes upon exposure to PFOA and PFBS at the highest concentration (4 to 1 lipid mixtures to PFAS molar ratio), with greater changes for PFOA than PFBS (as shown in Table 1). No such frequency shifts were observed in the case of DOPC/DOPG mixed membranes except upon exposure to PFOA. The observed increase in frequencies of both lipid mixtures in the presence of PFOA signify increasing intermolecular chain decoupling and disordering effect of PFOA on membranes formed from these lipid mixtures, with greater extent of such changes for lipid mixtures of DOPE/DOPG (3:1) compared to DOPC/DOPG (3:1). The vibrational spectra indicate an increase in acyl chain disorder, likely resulting from reduced lipid packing and a higher average surface area per lipid, which is consistent with our observation of increased water permeability. Note that our Raman spectroscopic data analysis of the CH region represents a bulk measurement, averaging across all regions within the sample.

**Table 1:** Raman-Active CH Stretching Vibration Frequencies (cm^–1^) of the Lipid Mixtures upon Exposure to PFOA and PFBS

lipid mixtures to PFAS molar ratio	DOPC/DOPG (3:1)	DOPE/DOPG (3:1)
control	2845 (ν_s_) and 2889 (ν_as_)	2847 (ν_s_) and 2889 (ν_as_)
PFOA (4 to 1)	2847 (ν_s_) and 2889 (ν_as_)	2849 (ν_s_) and 2894 (ν_as_)
PFBS (4 to 1)	2845 (ν_s_) and 2889 (ν_as_)	2847 (ν_s_) and 2892 (ν_as_)

## Conclusions

Biological membranes, whether in Gram-negative or Gram-positive bacteria, possess greater complexity, including diverse proteins, lipids, and structural components often absent in models. However, model membranes provide controlled environments for studying specific membrane properties, interactions with other molecules that would be challenging to isolate in live bacteria. This study provides a systematic evaluation of how model bacterial membranes with diverse lipid compositions respond to varying concentrations of long-chain PFOA and short-chain PFBS, highlighting the role of membrane lipid content in PFAS interactions. Our findings reveal that both compounds perturb membranes containing DOPC/DOPG and DOPE/DOPG, with effects varying by concentration and lipid components. Water permeability data provide evidence for lipid component-dependent membrane modifying characteristics, with DOPE-containing membranes showing greater susceptibility to PFAS exposure due to higher hydrogen bonding capability. DSC and vibrational spectroscopic studies corroborate these findings, indicating more pronounced effects in DOPE/DOPG mixtures compared to DOPC/DOPG. The differential interactions of PFOA and PFBS with membranes highlight the complex interplay of electrostatic interactions, van der Waals forces, and hydrogen bonding underlying PFAS-membrane interactions. The higher membrane/water partition coefficients of PFOA compared to PFBS emphasize the significance of the hydrophobic effect in long-chain versus short-chain PFAS-membrane interactions. Importantly, PFBS induced substantial structural modifications in bacterial model membranes at high concentrations, suggesting that replacing long-chain PFAS with shorter alternatives may not be as environmentally benign as anticipated. These findings underscore the importance of considering molecular structure and lipid composition in predicting PFAS environmental and health impacts. Recent studies have revealed a remarkable capacity of gut bacteria to bioaccumulate PFAS. Rapid bioaccumulation to a level of >50-fold enrichment of PFAS within bacterial pellets was observed, which they ascribe to intracellular PFAS concentration in the millimolar range.^20^ For human samples, a wide range of PFAS concentrations has been detected, typically from 10 to 10^4^ ng/mL (0.02 μM to 0.02 mM), varying by tissue and exposure source. Studies show PFAS distribution in the body is highly uneven, with some tissues accumulating much higher levels than blood.^10,73,74^ Given this, average measurements may underestimate localized accumulation due to heterogeneous distribution. Similarly to drug–membrane interactions, where local concentrations can well exceed tissue averages due to membrane heterogeneity,^75^ PFAS may also accumulate in specific human microenvironments at elevated levels. Our research contributes to a deeper understanding of how different lipid compositions sensitively respond to PFOA and PFBS exposure, modifying cell membranes at the molecular level. This knowledge is essential for improving bioremediation strategies and probing indirect effects on human health, emphasizing the need for comprehensive studies on diverse membranes and PFAS of varying chemical structures.

## Supplementary Material



## Notes

Analysis of water permeability data; water permeability coefficient for lipid mixtures as a function of PFOA or PFBS concentrations; DSC thermograms with curve-fitting simulations; DSC thermodynamic data; ATR-IR spectra in the stretching vibration of the acyl chain CH_2_ groups of DOPC/DOPG (3:1) model membranes; ATR-IR spectra in the stretching vibration of the carbonyl C=O bands of DOPE/DOPG (3:1) model membranes; ATR-IR spectra in the region of antisymmetric PO_2_^–^ stretching vibration bands and carbony C=O bands of DOPC/DOPG (3:1) and; the CH_2_ antisymmetric stretching vibration frequencies (IR) of the lipid mixtures upon exposure to PFOA and PFBS; Raman spectra of lipid mixtures with varying concentrations of PFOA or PFBS; Raman intensity ratios (*I*_2930_ /*I*_2845_) of CH stretching vibration peaks of the lipid mixtures upon exposure to PFOA and PFBS (PDF)
