# Supplementary material for: Membrane-Modifying Effects of Perfluoroalkyl Substances in Model Bacterial Membranes
Source: ACS Omega. 2025 Aug 26;10(35):39884–97. doi: 10.1021/acsomega.5c04177 (PMC12423796; doi:10.1021/acsomega.5c04177)
Supplement: Supplementary file 1 [file ao5c04177_si_001.pdf]

Supporting Information

Membrane-modifying effects of perfluoroalkyl substances in model bacterial membranes

*Micaela Panella, Amani Rabadi, Jasmin Ceja-Vega, Jessica Said, Elizabeth Andersen, Joseph Mitchell, Jacqueline Ceja, and Sunghee Lee**

Department of Chemistry and Biochemistry, Iona University, 715 North Avenue, New Rochelle, New York 10801, USA

*To whom correspondence should be addressed. Tel: 914-633-2638. E-mail: SLee@iona.edu

List of Supplemental Figures and Tables

Figure S1. Schematics of droplet interface bilayer (DIB) formation and osmotic water transport across DIB.

Figure S2. DSC thermograms with curve-fitting simulations.

Figure S3. Representative ATR-IR spectra in the stretching vibration of the acyl chain CH_2 groups of DOPC:DOPG (3:1) model membranes, with PFOA and PFBS at 25 °C.

Figure S4. Representative ATR-IR spectra in the stretching vibration of the carbonyl C=O bands of DOPE:DOPG (3:1) model membranes, with (A) PFOA and (B) PFBS at 25 °C.

Figure S5. Representative ATR-IR spectra in the region of antisymmetric PO_2^- stretching vibration bands and carbony C=O bands of DOPC:DOPG (3:1) with (A) PFOA and (B) PFBS.

Figure S6. Representative Raman spectra of DOPC:DOPG (3:1) with PFOA and PFBS.

Figure S7. Representative Raman spectra of DOPE:DOPG (3:1) with PFOA and PFBS.

Table S1. Water permeability coefficient at 30°C for DIB formed by DOPC:DOPG (3:1) and DOPE:DOPG (3:1), as a function of PFOA or PFBS concentratons.

Table S2. Thermodynamic parameters for main phase transition of MLVs of 3DOPC:1DOPG, and 3DOPE:1DOPG with varying PFOA and PFBS concentrations.

Table S3. The IR-active CH_2 antisymmetric stretching vibration frequencies (ν_{as}, cm^{-1}) of the lipid mixtures upon exposure to PFOA and PFBS.

Table S4. Raman intensity ratios (I_{2930}/ I_{2845}) of CH stretching vibration peaks of the lipid mixtures upon exposure to PFOA and PFBS.

Water Permeability Data Analysis

Water permeability was assessed using model membranes created through the droplet interface bilayer (DIB) technique. In this method, aqueous microdroplets surrounded by lipid monolayers are brought into contact, forming an interface that closely mimics the double-leaflet structure of biological cell membranes. This DIB serves as an effective model for studying membrane properties, including water permeability, in a controlled environment.

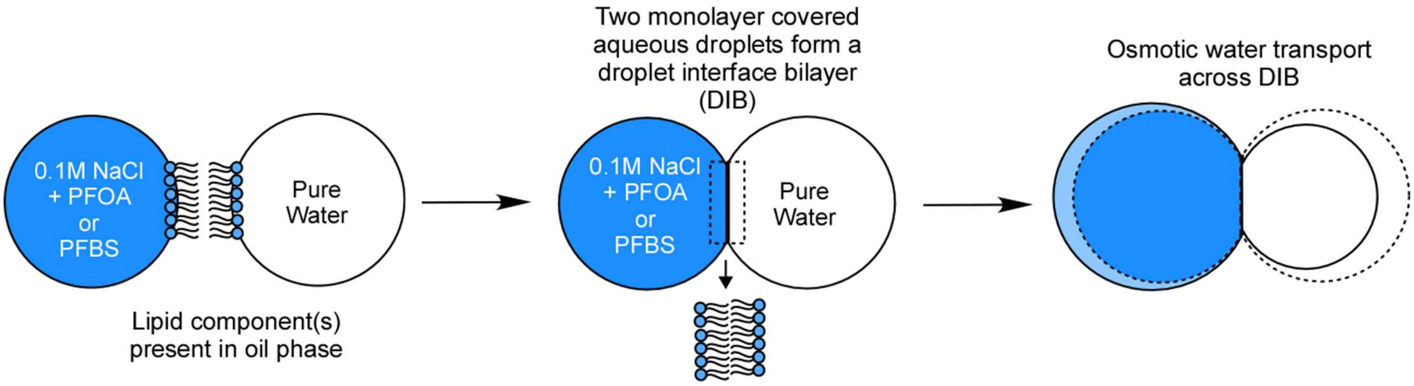

Figure S1. Schematics of droplet interface bilayer (DIB) formation and osmotic water transport across DIB. A circle with dotted line shows a swelling droplet (left) and shrinking droplet (right) upon osmotic water transport.

Figure S1 shows a schematic description of the DIB-based water permeability measurement system, designed to detect the effect of PFOA or PFBS. A pair of osmotically unbalanced aqueous droplets are used, one containing pure water and the other containing 0.1 M NaCl with varying concentrations of PFOA or PFBS (up to 3 mM). When two osmotically unbalanced microdroplets adhere to a bilayer, the osmotic gradient propels water transport through the droplet bilayer (as indicated by the arrow in Figure S1), leading to a noticeable change in droplet diameter. Any electrolyte flux is expected to be negligible compared to that of water, as ion permeation is typically almost eight orders of magnitude slower than that of water. The corresponding changes in droplet volume over time (dV/dt) is measured optically by microscopic observation; and the behavior of the system follows the expression of equation (1) based on Fick's Law:

$$\frac{dV(t)}{dt} = -P_f A(t) v_w \Delta C(t) \qquad (1)$$

where A is the geometric bilayer area, ν_w is the molar volume of water (18 mL/mol), **Δ**C(t) is the osmolality gradient between two droplets, and P_f is the bilayer permeability coefficient of water. The volume change with time (dV/dt) is related to the bilayer permeability coefficient of water, P_f, as expressed in the Equation (1). When the bilayer contact area is constant, the time evolution of the swelling droplet can be obtained from the following equation derived from the integration

of eqn. 1, with the following simplifying assumption: since one of the droplets (the shrinking droplet) contains no osmotic agent, its concentration does not change with time:[1, 2]

$$\left(\frac{V}{V_o}\right)^2 = \left(\frac{2P_f A v_w C_o}{V_o}\right)t + 1 \qquad (2)$$

Using the measured values for: initial size of the osmotic (swelling) droplet; bilayer contact area (A); and initial osmolarity of the osmotic droplet (C_o), then the coefficient P_f for bilayer water permeability may be derived from eqn. 2 from the slope of the curve obtained by plotting $(V/V_o)^2$ as a function of time. All data points presented in this paper are an average ($n \geq 50$) of individual permeability runs, each of which took place over a time course (~5 min) for osmotic water movement across the droplet bilayer, during which time the droplet contact area (A) remains constant. The recorded videos and images were post-analyzed to measure the dimension of droplets and contact area using custom built image analysis software. All droplet pairs had substantially the same initial size relative to each other, in the diameter range of 100 ± 5 μm diameter.

Table S1. Water permeability coefficient at 30°C for DIB formed by DOPC:DOPG (3:1) and DOPE:DOPG (3:1), as a function of PFOA or PFBS concentratons. Each data point represents an average of individual permeability runs ($n \geq 50$), and standard deviation as error bars. Statistical comparisons between each concentration and the control (*), as well as between successive concentration values (†), were performed using t-tests. A p values < 0.05 were considered statistically significant, and signficance is indicated in the table.

PFOA or PFBS (mM)	Water permeability coefficient (μm/s)			
	DOPC:DOPG (3:1)		DOPE:DOPG (3:1)	
	PFOA	PFBS	PFOA	PFBS
0	62 ± 5	62 ± 5	58 ± 4	58 ± 4
0.1	63 ± 5	60 ± 7	63 ± 5*	60 ± 5*
0.5	64 ± 8	61 ± 6	73 ± 4*†	62 ± 6*
1.0	65 ± 6*	66 ± 5*†	75 ± 5*†	63 ± 4*
2.0	68 ± 4*†	67 ± 6*	76 ± 3*	65 ± 7*
3.0	67 ± 9*	69 ± 8*	77 ± 2*	66 ± 4*

*$p < 0.05$ vs. control
†$p < 0.05$ vs. between successive concentration

[1] Lopez, M.; Evangelista, S. E.; Morales, M.; Lee, S. Enthalpic effects of chain length and unsaturation on water permeability across droplet bilayers of homologous monoglycerides. *Langmuir* 2017, 33 (4), 900-912.
[2] Thiam, A. R.; Bremond, N.; Bibette, J. From stability to permeability of adhesive emulsion bilayers. *Langmuir* 2012, 28 (15), 6291-6298.

Thermodynamic data

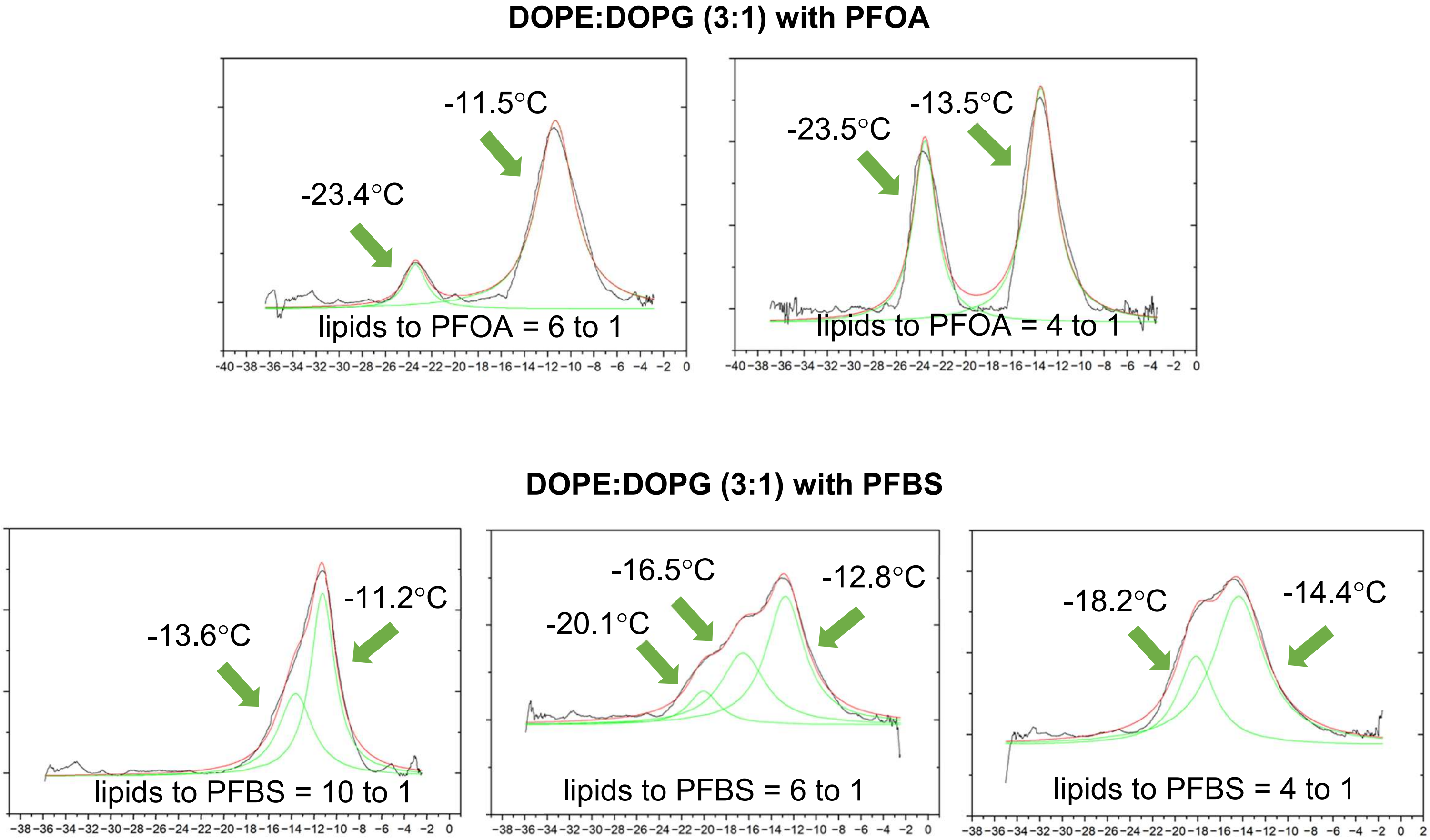

Figure S2. Curve-fitting simulations were performed using Origin software to deconvolute the data into multiple components, each corresponding to distinct T_m regions.

Table S2.

A. Thermodynamic parameters for main phase transition of MLVs of 3DOPC:1DOPG, with varying PFOA and PFBS concentrations.

Lipids: PFOA or PFBS (mol ratio)	PFOA			PFBS		
	T_m (°C)	ΔH (kcal/mol)	FWHM (°C)	T_m (°C)	ΔH (kcal/mol)	FWHM (°C)
1:0 (control)	–16.67 ± 0.20	8.35 ± 0.20	0.46 ± 0.01	–16.77± 0.30	8.66 ± 0.22	0.52 ± 0.01
100:1	–16.61 ± 0.31	7.98 ± 0.64	1.43 ± 0.01	–16.82 ± 0.25	8.35 ± 0.48	1.96 ± 0.01
20:1	–17.15 ± 0.42	6.61 ± 0.41	2.32 ± 0.01	–16.95 ± 0.43	7.86 ± 0.96	2.78 ± 0.02
10:1	–17.04 ± 0.36	5.98 ± 0.72	4.18 ± 0.04	–16.45 ± 0.36	6.83 ± 0.80	4.32 ± 0.03
6:1	–17.32 ± 0.62	4.85 ± 0.95	4.56 ± 0.04	–16.34 ± 0.22	6.07 ± 0.74	3.17 ± 0.02
4:1	–17.36 ± 0.38	2.86 ± 0.78	4.17 ± 0.05	–16.47 ± 0.35	4.52 ± 0.86	3.46 ± 0.02

B. Thermodynamic parameters for main phase transition of MLVs of 3DOPE:1DOPG, with varying PFOA and PFBS concentrations.

Lipids: PFOA or PFBS (mol ratio)	PFOA			PFBS		
	T_m (°C)	ΔH (kcal/mol)	FWHM (°C) or other peak, T_m* (rel area %)	T_m (°C)	ΔH (kcal/mol)	FWHM (°C) or other peak, T_m* (rel area %)
1:0 (control)	–8.14 ± 0.43	8.01 ± 0.15	1.06 ± 0.01	–8.84 ± 0.32	7.11 ± 0.22	2.04 ± 0.01
100:1	–8.12 ± 0.21	7.21 ± 0.62	1.21 ± 0.01	–9.03 ± 0.18	7.03 ± 0.18	2.85 ± 0.02
20:1	–9.22 ± 0.18	5.80 ± 0.51	2.18 ± 0.01	–10.00 ± 0.24	6.75 ± 0.57	4.10 ± 0.03
10:1	–10.29 ± 0.26	4.85 ± 0.43	3.70 ± 0.02	–11.24 ± 0.41	5.68 ± 0.45	–13.64 (38%)
6:1	–11.46 ± 0.44	3.03 ± 0.85	–23.40 (13%)	–12.75 ± 0.27	4.52 ± 0.83	–16.53 (35%); –20.05 (12%)
4:1	–13.49 ± 0.83	2.57 ± 0.74	–23.54 (39%)	–14.39 ± 0.53	3.15 ± 0.59	–18.16 (31%)

*Curve-fitting simulations were performed using Origin software to deconvolute and fit into two or more components corresponding to lower T_m and higher T_m regions (Figure S2 in the Supporting Information). Enthalpy data represents the total area for all peaks.

ATR-IR Data

Table S3. The IR-active CH_2 antisymmetric stretching vibration frequencies (ν_{as}, cm^{-1}) of the lipid mixtures upon exposure to PFOA and PFBS.

Lipid mixtures to PFAS molar ratio	DOPC:DOPG (3:1)		DOPE:DOPG (3:1)	
	PFOA	PFBS	PFOA	PFBS
control	2923.6 ± 0.5	2923.6 ± 0.5	2923.6 ± 0.5	2923.6 ± 0.5
500 to 1	2923.6 ± 0.5		2923.6 ± 0.5	2923.6 ± 0.5
100 to 1		2923.6 ± 0.5		2923.6 ± 0.5
50 to 1	2923.6 ± 0.5	2923.6 ± 0.5	2924.0 ± 0.5	2923.6 ± 0.5
30 to 1	2923.6 ± 0.5	2923.6 ± 0.5	2924.0 ± 0.5	
20 to 1	2924.0 ± 0.5	2923.6 ± 0.5	2924.0 ± 0.5	2924.0 ± 0.5
10 to 1	2924.0 ± 0.5	2924.0 ± 0.5	2924.5 ± 0.5	2924.0 ± 0.5
4 to 1	2924.5 ± 0.5	2924.0 ± 0.5	2925.0 ± 0.5	2924.5 ± 0.5

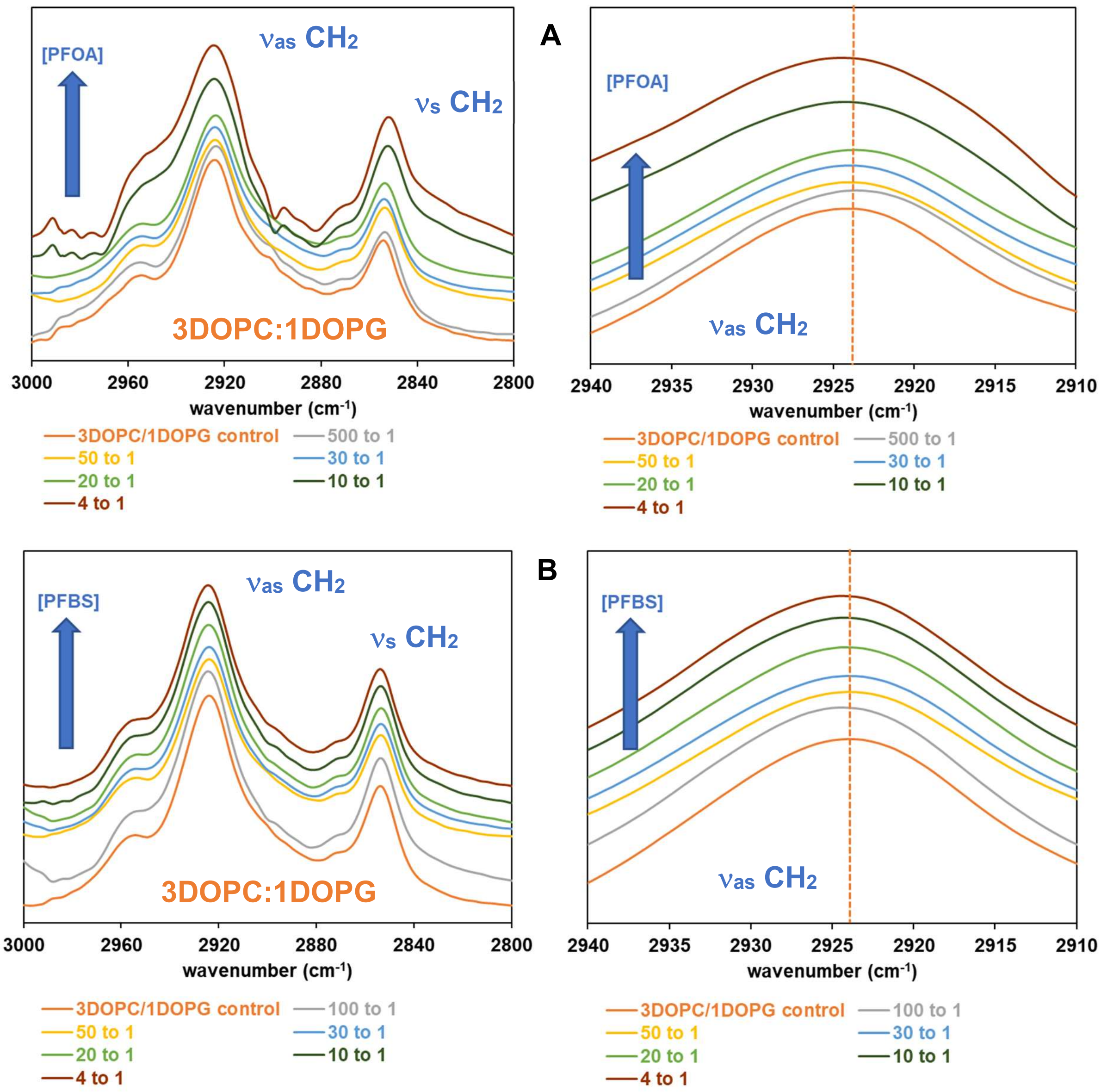

Figure S3. Representative ATR-IR spectra in the stretching vibration of the acyl chain CH_2 groups of DOPC:DOPG (3:1) model membranes, with increasing concentrations (molar ratio of lipid to PFAS) of (A) PFOA and (B) PFBS at 25 °C, with the expanded regions of ν_{as} CH_2. A vertical dotted line indicates the position of the ν_{as} CH_2 band in the control spectrum.

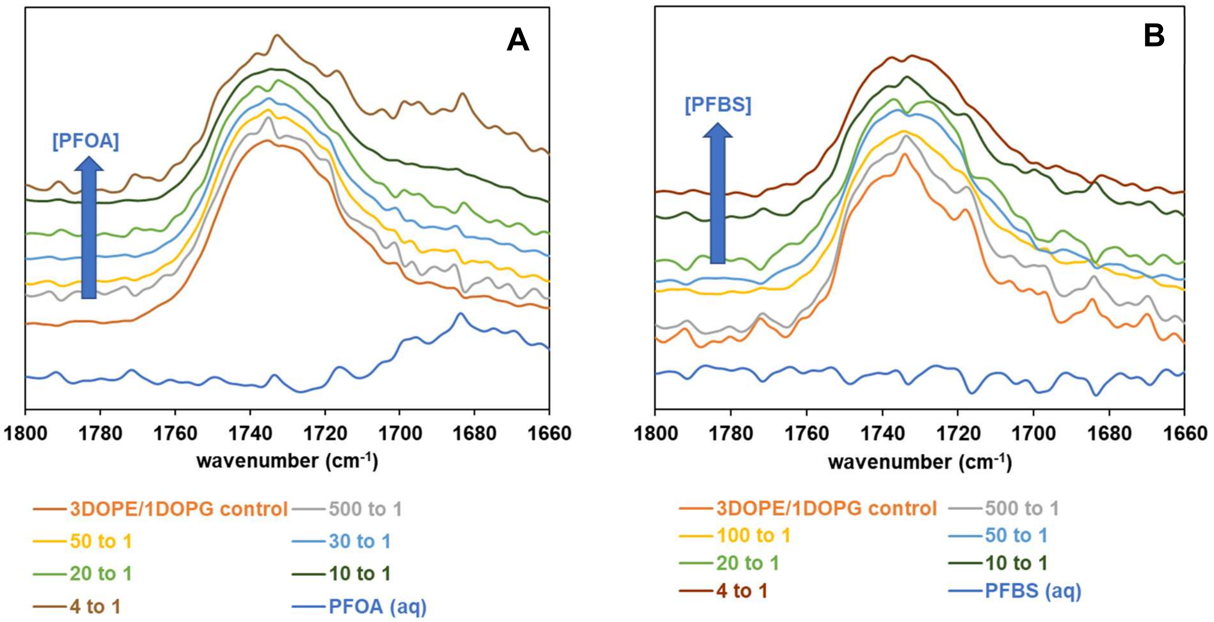

Figure S4. Representative ATR-IR spectra in the stretching vibration of the carbonyl C=O bands of DOPE:DOPG (3:1) model membranes, with increasing concentrations (molar ratio of lipid to PFAS) of (A) PFOA and (B) PFBS at 25 °C. The blue trace at the bottom of each panel represents the ATR-IR spectrum of PFOA or PFBS in aqueous solution..

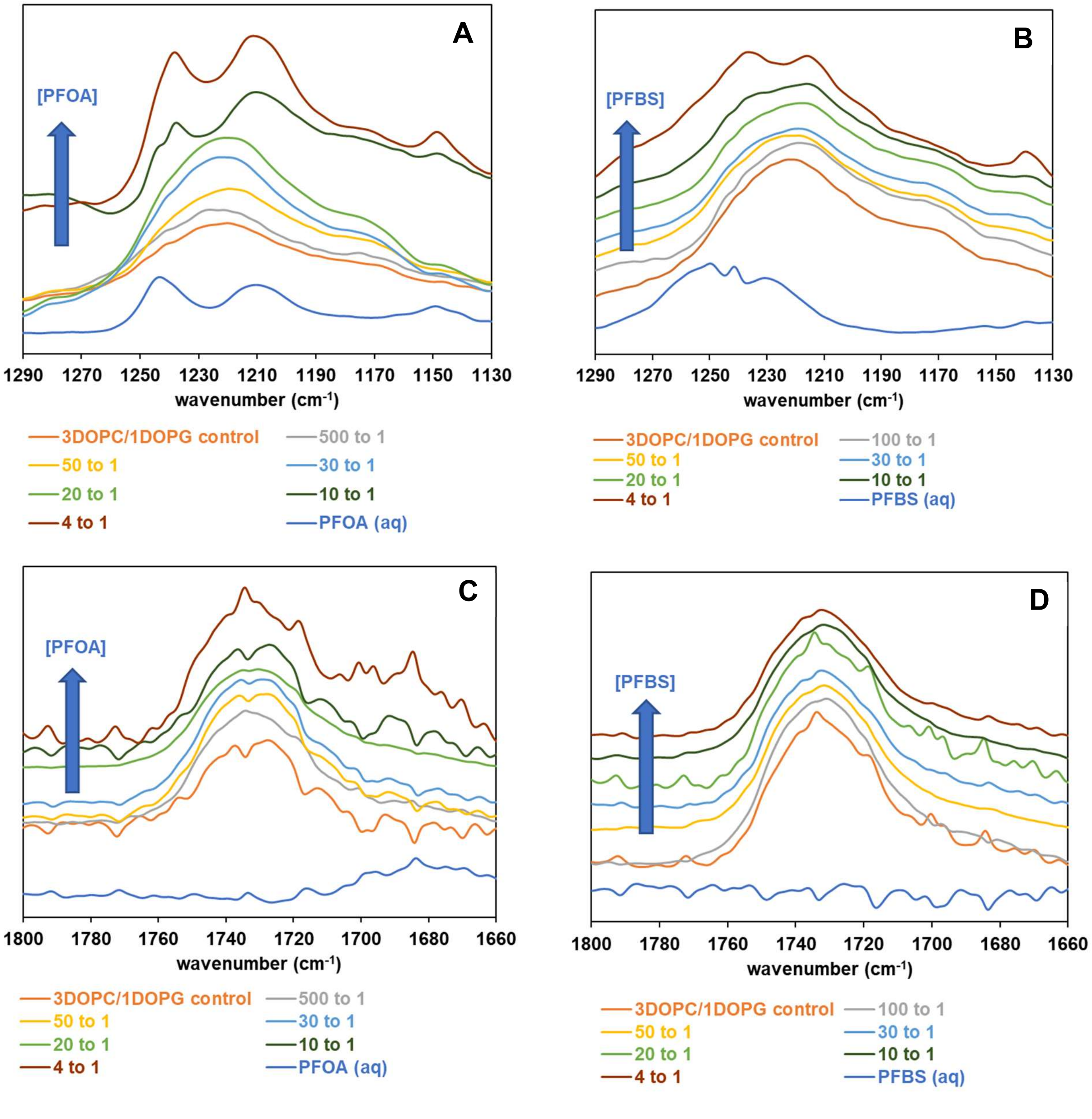

Figure S5. Representative ATR-IR spectra in the region of (A and B)antisymmetric PO_2^- stretching vibration bands and (C and D) carbony C=O bands of DOPC:DOPG (3:1) with varying mol ratios of PFOA and PFBS. The blue trace at the bottom of each panel represents the ATR-IR spectrum of PFOA or PFBS in aqueous solution.

Raman spectroscopic data

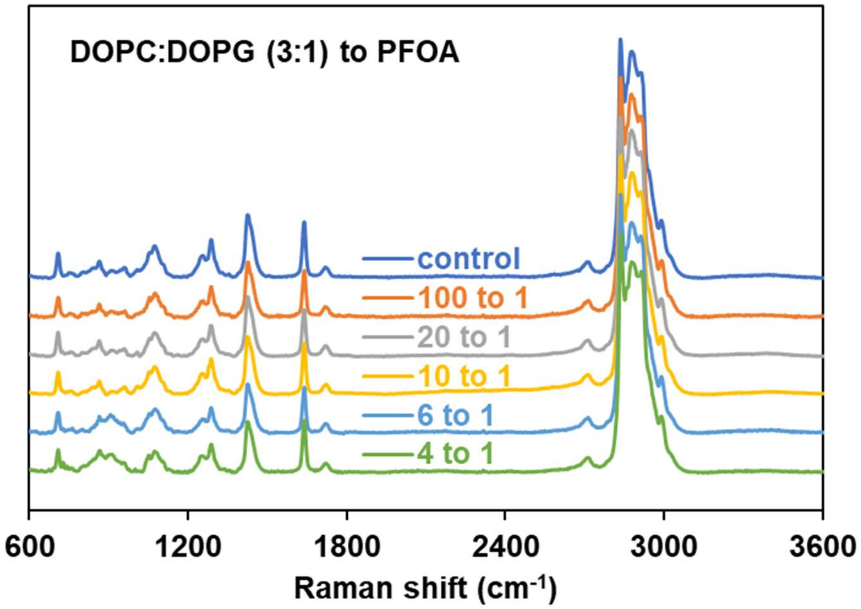

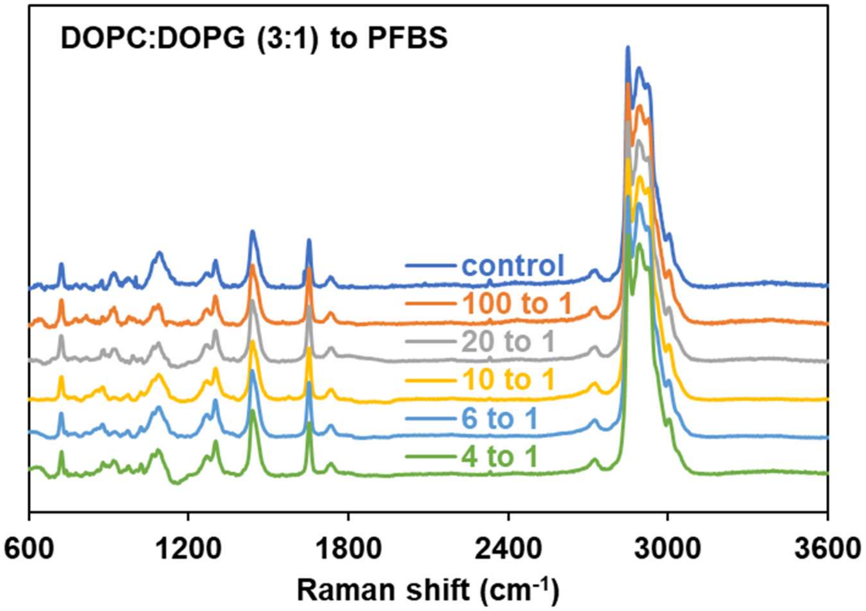

Figure S6. Representative Raman spectra of DOPC:DOPG (3:1) at various concentrations of PFOA and PFBS at ambient temperature. Spectra are normalized to the intensity ~2850 cm^{-1} (the most intense peak) for comparison, and vertically shifted for clarity.

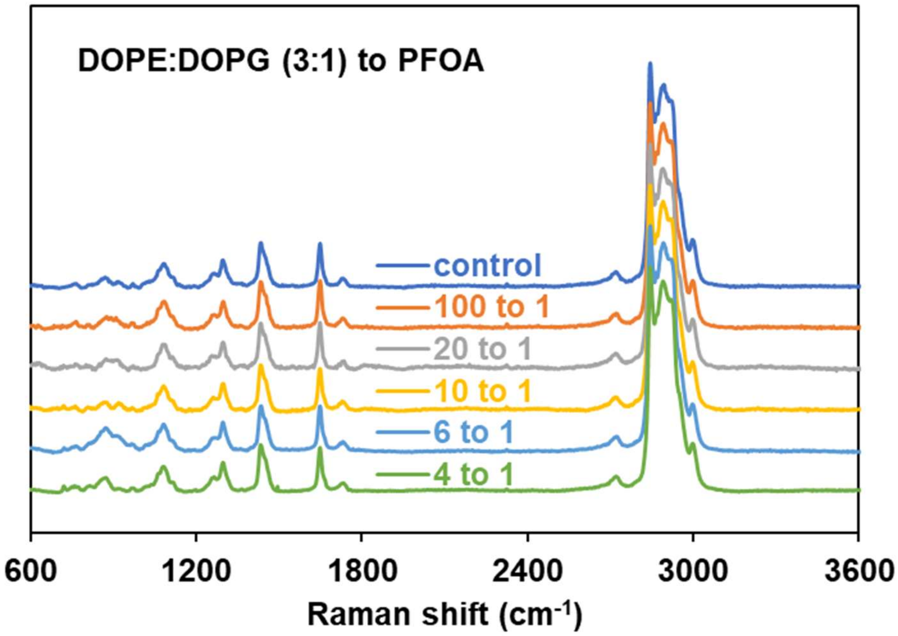

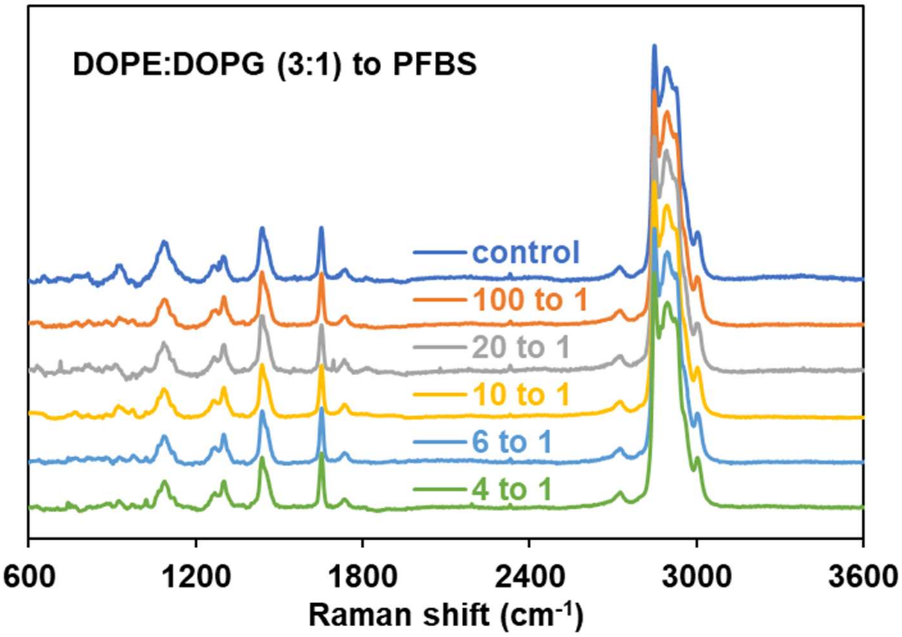

Figure S7. Representative Raman spectra of DOPE:DOPG (3:1) at various concentrations of PFOA and PFBS at ambient temperature. Spectra are normalized to the intensity ~2850 cm^{-1} (the most intense peak) for comparison, and vertically shifted for clarity.

Table S4. Raman intensity ratios (I_{2930}/ I_{2845}) of CH stretching vibration peaks of the lipid mixtures upon exposure to PFOA and PFBS

Lipid mixtures to PFAS molar ratio	DOPC:DOPG (3:1)		DOPE:DOPG (3:1)	
	PFOA	PFBS	PFOA	PFBS
control	0.836 ± 0.005	0.836 ± 0.005	0.834 ± 0.004	0.834 ± 0.004
100 to 1	0.835 ± 0.006	0.836 ± 0.005	0.833 ± 0.003	0.834 ± 0.004
20 to 1	0.834 ± 0.004	0.834 ± 0.003	0.837 ± 0.005	0.835 ± 0.003
10 to 1	0.835 ± 0.003	0.838 ± 0.006	0.850 ± 0.007	0.835 ± 0.004
6 to 1	0.838 ± 0.008	0.840 ± 0.004	0.853 ± 0.008	0.839 ± 0.007
4 to 1	0.844 ± 0.007	0.842 ± 0.003	0.856 ± 0.006	0.841 ± 0.003
